# A novel tyrosine tRNA-derived fragment, tRF^Tyr^, induces oncogenesis and lactate accumulation in LSCC by interacting with LDHA

**DOI:** 10.1186/s11658-023-00463-8

**Published:** 2023-06-26

**Authors:** Rui Zhao, Zhenming Yang, Bo Zhao, Wenjing Li, Yaohui Liu, Xiaoxue Chen, Jing Cao, Jiarui Zhang, Yan Guo, Licheng Xu, Jinpeng Wang, Yanan Sun, Ming Liu, Linli Tian

**Affiliations:** 1 Department of Otolaryngology-Head and Neck Surgery, The Second Affiliated Hospital of Harbin Medical University, Harbin, China; 2 School of Forestry, Northeast Forestry University, Harbin, China

**Keywords:** tsRNAs, tRF^Tyr^, LDHA, Lactate, LSCC

## Abstract

**Background:**

Transfer (t)RNA-derived small RNA (tsRNA), generated from precursor or mature tRNA, is a new type of small non-coding RNA (sncRNA) that has recently been shown to play a vital role in human cancers. However, its role in laryngeal squamous cell carcinoma (LSCC) remains unclear.

**Methods:**

We elucidated the expression profiles of tsRNAs in four paired LSCC and non-neoplastic tissues by sequencing and verified the sequencing data by quantitative real-time PCR (qRT–PCR) of 60 paired samples. The tyrosine-tRNA derivative tRF^Tyr^ was identified as a novel oncogene in LSCC for further study. Loss-of-function experiments were performed to evaluate the roles of tRF^Tyr^ in tumorigenesis of LSCC. Mechanistic experiments including RNA pull-down, parallel reaction monitoring (PRM) and RNA immunoprecipitation (RIP) were employed to uncover the regulatory mechanism of tRF^Tyr^ in LSCC.

**Results:**

tRF^Tyr^ was significantly upregulated in LSCC samples. Functional assays showed that knockdown of tRF^Tyr^ significantly suppressed the progression of LSCC. A series of mechanistic studies revealed that tRF^Tyr^ could enhance the phosphorylated level of lactate dehydrogenase A (LDHA) by interacting with it. The activity of LDHA was also activated, which induced lactate accumulation in LSCC cells.

**Conclusions:**

Our data delineated the landscape of tsRNAs in LSCC and identified the oncogenic role of tRF^Tyr^ in LSCC. tRF^Tyr^ could promote lactate accumulation and tumour progression in LSCC by binding to LDHA. These findings may aid in the development of new diagnostic biomarkers and provide new insights into therapeutic strategies for LSCC.

**Supplementary Information:**

The online version contains supplementary material available at 10.1186/s11658-023-00463-8.

## Background

Laryngeal cancer, as the second most prevalent malignant tumour occurring in the head and neck region, has become a serious threat to human health worldwide, especially in Asia [1]. It mainly originates from squamous cells. Laryngeal squamous cell carcinoma (LSCC) accounts for 95% of laryngeal cancer cases [2]. Although the current main treatments for LSCC, including surgical resection and adjuvant radio-chemotherapy, have improved the cure rate of early-stage LSCC patients, the overall 5-year survival rate remains dismal [3]. Hence, it is critical to explore potential new biomarkers and therapeutic targets to improve the prognosis of LSCC.

Transfer RNAs (tRNAs), the most abundant small non-coding RNAs, account for 4–10% of the total RNAs in cells [4]. tRNAs are transverters in the translation machinery that deliver amino acids to the ribosome and decode messenger RNA (mRNA) into the corresponding polypeptide chain. Through in-depth analysis of sequencing data, emerging non-coding small RNAs called tRNA-derived small RNAs (tsRNAs) have been identified as dynamic regulators with various biological functions. These tRNA derivatives are divided into two types according to their cleavage at different positions in both mature and precursor tRNAs: tRNA-derived stress-induced RNA (tiRNA, 28–36 nucleotides in length) and tRNA-derived fragments (tRFs, 14–30 nucleotides in length) [5]. tiRNAs comprise two subclasses: 5′-tRNA halves (tiRNA-5) and 3′-tRNA halves (tiRNA-3) of mature tRNA cleaved in the anti-codon loop by the ribonuclease angiogenin [5]. tRFs are derived from mature tRNA or precursor tRNAs and are divided into four subclasses depending on their original sites in the pretRNA or mature tRNA: tRF-5, tRF-3, tRF-1 and i-tRF [5]. The regulatory mechanisms of tsRNAs identified to date are as follows: (1) they regulate mRNA stability, similar to miRNAs [6]; (2) they inhibit the initiation and extension of translation [7]; (3) they regulate ribosome biogenesis [8]; and (4) they function as a new type of epigenetic factor to change the spatial conformation of proteins to affect their function, such as DNA methylation, histone modification and chromatin remodelling [9]. Growing evidence has indicated that tsRNAs can regulate gene expression at both the transcriptional and post-transcriptional levels in a variety of human diseases, such as inflammatory diseases, metabolic diseases and cancers. As reported previously, tumour cells absorb abundant proteins for rapid growth, which promotes the formation of a microenvironment with limited oxygen and nutrients [8, 10]. Tumour cells adapt to stressful environments by regulating tsRNA expression and tsRNA have been found to be dysregulated in a variety of malignancies, including breast cancer [11], multiple myeloma [12], bladder cancer [13] and prostate cancer [14]. A recent review in the Journal of Hematology and Oncology confirmed the crucial role of tRFs in tumour diagnosis and treatment [15]. However, there have been few systematic evaluations of tsRNAs in LSCC, and their potential role requires further investigation. tRF^Tyr^, a significantly upregulated tsRNA in LSCC tissues, attracted our attention for further exploration. Functional experiments indicated that tRF^Tyr^ downregulation inhibited tumorigenesis and metastasis in LSCC. A mechanistic study demonstrated that tRF^Tyr^ could bind to lactate dehydrogenase A (LDHA), the key enzyme for pyruvate-to-lactate conversion. We then measured the activity and expression levels of LDHA. The data showed that knockdown of tRF^Tyr^ attenuated LDHA activity and lactate production in LSCC cells in vitro and in vivo. Thus, our study revealed that tRF^Tyr^, a novel oncogene, could induce lactate accumulation and promote tumorigenesis in LSCC, and this finding may provide new therapeutic targets for LSCC.

## Methods

### Tissue samples

Sixty patients with LSCC were enrolled in the study. Each pair of LSCC tissues and corresponding non-neoplastic tissues was collected from the same patient who was initially diagnosed with LSCC and underwent surgical resection without any pre-operative adjuvant therapy at the Second Affiliated Hospital of Harbin Medical University. Four pairs of tissues were used for tsRNA sequencing analysis. The tissues were snap frozen in liquid nitrogen within 15 min after excision or fixed in formalin for paraffin-embedded specimens.

### RNA isolation, pre-treatment and sequencing

Total RNA from LSCC and matched peritumour tissues was isolated using TRIzol reagent (Invitrogen, USA) and quantified using a NanoDrop-1000 (NanoDrop Technologies, Rockland, DE, USA). The details of RNA quantification and quality assurance by NanoDrop ND-1000 are described in Additional file 1: Table S1. Total RNA samples were pre-treated to remove RNA modifications that interfered with small RNA-sequencing library construction. The details are as follows: 3′-aminoacyl (charged) deacylation to obtain 3′-OH for 3′-adaptor ligation, 3′-cP (2′,3′-cyclic phosphate) removal to obtain 3′-OH for 3′-adaptor ligation, 5′-OH (hydroxyl group) phosphorylation to obtain 5′-P for 5′-adaptor ligation and m1A and m3C demethylation for efficient reverse transcription. The pre-treated total RNA was sequentially ligated to 3′ and 5′ small RNA adapters. cDNA was synthesized and amplified using proprietary Illumina RT primers and amplification primers. Size-selected PCR-amplified fragments of 134–160 bp were extracted and quantified using an Agilent 2100 Bioanalyzer (Invitrogen). The completed libraries were denatured and diluted for sequencing on an Illumina NextSeq 500 system (Illumina) according to the manufacturer’s instructions. The DNA fragments in well-mixed libraries were denatured with 0.1 M NaOH to generate single-stranded DNA molecules and loaded onto a reagent cartridge at a concentration of 1.8 pM. Sequencing was performed on a NextSeq system using a NextSeq 500/550 V2 kit (#FC-404-2005, Illumina) according to the manufacturer’s instructions. Sequencing was performed for 50 cycles.

### tsRNA sequencing data analysis

Solexa pipeline v1.8 (Off-Line Base Caller software, v1.8) was used for image analysis and base calling. Raw sequencing data generated by an Illumina NextSeq 500 were evaluated for quality using FastQC software [16], and the quality score plot of each sample is shown in Additional file 1: Fig. S1. A summary of the quality scores is presented in Additional file 1: Table S2. After quality control, the 5′ and 3′ adapter sequences were trimmed from the clean reads using cutadapt [17], and reads with lengths shorter than 14 nt or longer than 40 nt were discarded. Trimmed reads (trimmed 5′ and 3′ adaptor bases) were aligned, allowing for only one mismatch with the mature tRNA sequence, and then reads that did not map were aligned, allowing for only one mismatch with precursor tRNA sequences, with Bowtie software [18]. The expression profiles of tsRNAs were determined based on the number of reads mapped. The mapping summary is presented in Additional file 1: Table S3. The tsRNAs were then filtered using the count value in the R package edgeR [19]. tsRNAs with a fold change ≥ 1.5 and *P*-value < 0.05 were identified as significantly dysregulated.

### Real-time PCR validation, western blotting and immunohistochemical (IHC) analysis

Real-time PCR was performed to confirm and verify the sequencing data. Total RNA isolated from 60 paired LSCC samples was reverse transcribed to cDNA using the rtStar™ tRF&tiRNA Pretreatment Kit (Arraystar, MD, USA) and rtStar First-Strand cDNA Synthesis Kit (3′ and 5′ adaptors) (Arraystar, MD, USA) according to the manufacturer’s instructions. PCR was performed using 2× PCR master mix (Arraystar, MD, USA) and conducted in a ViiA 7 Real-time PCR System (Applied Biosystems, CA, USA), including incubation at 95 °C for 10 min, followed by 40 cycles of 95 °C for 10 s, 60 °C for 60 s and 70 °C for 10 s. Details of the selected tRF transcripts are provided in Additional file 1: Table S4. The dissolution curves for each tRF transcript during real-time PCR are shown in Additional file 1: Fig. S2A. The expression of LDHA in tumour tissues was evaluated using IHC, as described in Additional file 1: Method S1. Western blot analysis was performed as previously described in Additional file 1: Methods S2 [20, 21]. The films were scanned, and the optical density of each band was determined. Representative raw data (not cut) are shown in Additional file 1: Fig. S2B.

### Bioinformatic analysis

The reads were mapped using the GtRNAdb: Genomic tRNA Database (http://gtrnadb.ucsc.edu/) [22]. Considering that tsRNAs scan for target RNAs similar to miRNAs, we predicted the target genes of the selected tsRNAs based on the TargetScan and Miranda algorithms. After submitting the tRF sequences into these tools, we synthesized the best predictions from each database to minimize their error rates. We evaluated the expression and target genes of tsRNAs using the ‘OncotRF’ and ‘MINTbase’ databases. Gene expression profiling interactive analysis 2 (GEPIA2), based on TCGA and GTEx data, was performed to observe the expression of LDHA in head and neck squamous cancer (HNSC). *P*-values below the cut-off of 0.05 were considered to indicate significant differences.

### Cell culture and transfection

AMC-HN8 (RRID: CVCL_5966) and TU212 (RRID: CVCL_4915) human LSCC cells [20, 23–25] were kindly provided by the BeNa Culture Collection (Jiangsu, China). Cells were cultured in high glucose Dulbecco’s modified Eagle medium (DMEM) with 10% foetal bovine serum (PAN-Biotech, Adenbach, Germany) and incubated in a humidified 37 °C incubator with 5% CO_2_. The tRF^Tyr^ shRNA lentiviral vector (GV280-EGFP) was purchased from GeneChem (Shanghai, China). An shRNA control with a non-targeting sequence was used as the control vector. The shRNA sequences used are listed in Additional file 1: Table S5. A total of 2 × 10^5^ cells/well were seeded in six-well plates overnight to transduce the lentiviral vector. The cells were incubated in a lentivirus-containing medium (10^8^ TU/mL) supplemented with 4 μg/mL polybrene for 24 h. At 72 h post-transfection, stably transfected cells were selected using 2.0 μg/mL puromycin for 2 weeks.

### RNA pull-down experiment

The cells were added to 1000 μL of working solution (Phosphate buffered saline+1%Nonidet P-40 (PBSN) lysate mixed with a protease inhibitor). The cell lysate was then sonicated in ice-cold water. Proteins (2 mg) were obtained for the RNA pull-down experiment. Beads were washed six times with wash buffer for 1 min per wash. Two hundred microlitres of buffer was added to resuspend the beads, 1 nM RNA was added and the sample was centrifuged for 15 min. The beads were washed again with wash buffer. Two milligrams of protein obtained in the previous test step was mixed with beads and centrifuged at low speed for 3 h. The supernatant was then transferred to a new Eppendorf tube and stored at −80 °C. Then, 500 μL of PBSN (DEPC-treated) was added, the sample was centrifuged at low speed for 2 min and the supernatant was discarded; this was repeated six times. Next, 100 µL elution buffer and 20 U benzonase were added, and the protein was eluted at 37 °C for 30 min. The supernatant was then transferred to a new low-binding Eppendorf tube. The beads were washed once with 100 µL of elution buffer, and the two supernatants were combined. The protein was precipitated with 0.1% sodium deoxycholate (SDC) and 10% trichloroacetic acid (TCA) at 4 °C for 2 h. The pellets were washed with precooled 80% acetone three times. Next, 5 mM tris-(2-carboxyethyl)-phosphine hydrochloride (TCEP) was added to each sample, followed by incubation with mixing at 55 °C for 10 min. Then, 10 mM iodoacetamide (IAA) was added after sample cooling, and the samples were incubated in the dark for 15 min. The sample was resuspended in 0.5 µg/µL trypsin and incubated at 37 °C overnight. The reaction was quenched with 1% TFA, and SDC was sedimented. The solution was centrifuged, and the supernatant was collected. The sediment was washed with 1% TFA, and the two supernatants were combined. Peptide desalting was performed for further liquid chromatography–mass spectrometry (LC–MS). For each sample, 5 μL peptides were separated and analysed using nano-UPLC (EASY-nLC1200) coupled to Q-Exactive mass spectrometry (Thermo Finnigan). Separation was performed using a reverse-phase column (100 µm, ID × 15 cm, Reprosil-Pur 120 C18-AQ, 1.9 µm, Dr. Math). The mobile phases were H_2_O with 0.1% formic acid (FA) and 2% acetonitrile (ACN) (phase A) and 80% ACN and 0.1% FA (phase B). Each sample was separated using a 120 min gradient at a flow rate of 300 nL/min. Gradient B was applied as follows: 8% to 35% for 92 min, 35% to 45% for 20 min, 45% to 100% for 2 min, 100% for 2 min, 100% to 2% for 2 min and 2% for 2 min. Raw MS files were processed using MaxQuant (version 1.5.6.0).

### LC–MS analysis

Each sample was mixed with 500 µL of 70% methanol aqueous solution, vortexed for 3 min, placed in a high-throughput tissue lysis instrument and shaken for 1 min at 50 Hz. After centrifugation at 12,000 RPM and 4 °C for 10 min, the supernatant was transferred to an injection vial through a film. Chromatographic conditions: ACQUITY UPLC® BEH Amide column (2.1 × 100 mm, 1.7 μm, Waters Corporation, USA); sample size, 2 μL; column temperature, 40 °C; mobile phase A-water (containing 10 mM ammonium acetate, 0.3% ammonia); B-acetonitrile (containing 90% acetonitrile/water). The mass spectrometry conditions were as follows: electrospray ionization (ESI) source, positive ionization mode and negative ionization mode. The scans were performed using declustering potential (DP) and collision energy (CE). All the samples were quantitatively analysed according to the established method of sample pre-treatment and instrumental analysis. Activity was estimated as the ratio of lactate to pyruvate.

### Parallel reaction monitoring (PRM)

PRM is an ion-monitoring technique that is performed on a mass spectrometer with high resolution and high mass accuracy. It can selectively quantify target proteins and peptides and provide relative or absolute quantification results [26]. For each sample, approximately 1/2 of the peptides were separated and analysed using nano-UPLC (EASY-nLC1200) coupled to a Q-Exactive mass spectrometer (Thermo Finnigan). The separation was performed using a reverse-phase column. Data-dependent acquisition was performed in profile and positive mode with an Orbitrap analyser at a resolution of 70,000 (at 200 *m*/*z*) and a *m*/*z* range of 350–1600 for MS1. For MS2, the resolution was set to 17,500, with a dynamic initial mass. Raw PRM data were processed using Skyline. The protein sequence database was downloaded from UNIPROT.

### RNA immunoprecipitation (RIP) assay

RIP was conducted with a Magna RIP kit (EMD Millipore, Billerica, MA, USA) according to the manufacturer’s instructions. TU212 cells were harvested and lysed with radioimmunoprecipitation assay lysis buffer. The lysates were incubated overnight with magnetic beads conjugated with LDHA antibody (ab52488; Abcam) or negative control immunoglobulin (Ig)G antibody (ab172730; Abcam) on a rotator at 4 °C. Immunoprecipitated RNA was isolated and enriched to detect tRF^Tyr^ by qRT‒PCR.

### Cell viability assay

Cell viability was evaluated using a cell counting kit 8 (CCK-8, Sigma Aldrich, MO, US), colony formation assay, and 5-ethynyl-2′-deoxyuridine assay (Edu; RiboBio, Guangzhou, China) according to the manufacturer’s instructions. For the CCK-8 assay, 10 μL of CCK-8 was added to each well of a 96-well plate at a concentration of 2 × 10^3^ cells/well. The absorbance was quantified at 450 nm using a microplate reader (Bio-Rad, Richmond, CA, USA). For the colony formation assay, the indicated cells were seeded in six-well plates (1000 cells/well). After 2 weeks of incubation, the colonies were stained with crystal violet and counted. For the EdU assay, the cells were incubated with EdU (RiboBio) for 2 h and washed twice with PBS. The cells were fixed with 4% paraformaldehyde for 30 min and stained with Apollo 643. After staining with Hoechst 33342 for 30 min, images were obtained using an ImageXpress high-content screening system (Molecular Devices).

### Transwell assay

Cell migration and invasion abilities were assessed using a Transwell chamber (BD Biosciences, San Jose, CA) according to the manufacturer’s instructions. A total of 1 × 10^4^ cells were added to the upper compartment of the Transwell chamber (24 well, 8 μm pores) and incubated at 37 °C for 24 h. Polycarbonate membranes with or without Matrigel (BD Biosciences) served as a barrier for invasion or migration assays. The cells that crossed the membrane were then fixed with 4% paraformaldehyde and stained with 0.1% crystal violet. The number of stained cells was counted in five individual fields at 200× magnification.

### Tumour model in vivo

Five-week-old male BALB/c nude mice from Vital River Laboratories (Beijing, China) were injected subcutaneously in the neck area with 100 μL of a suspension of stably transfected cells at a concentration of 2 × 10^8^ TU212 human LSCC cells to establish xenografts and divided into two groups (*n* = 6/group). The mice were euthanized for tumour evaluation after 5 weeks. Xenograft volume was calculated using the following formula: length × width^2^ × 0.5. The in vivo experiments were approved by the Animal Ethics Committee of Harbin University and performed according to the National Institute of Health Guide for the Care and Use of Laboratory Animals.

### Statistical analysis

Data are presented as the mean ± SD of three independent experiments. The difference in measurable variants between the two groups was assessed using Student’s *t*-test. All assays were performed in triplicate. Pearson’s correlation analysis was performed to determine the expression correlation between LDHA and tRF^Tyr^. The chi-square test was used to analyse the relationship between the expression of the indicators (tRF^Tyr^, LDHA and lactate) and clinicopathological characteristics. Statistical analysis was conducted using the GraphPad Prism software package (v. 6.0; San Diego, CA, USA) or SPSS software (version 20.0; SPSS, Chicago, IL, USA). Statistical significance was set at *P* < 0.05.

## Results

### Overview of tsRNA profiling in LSCC tissues

**Fig. 1:**
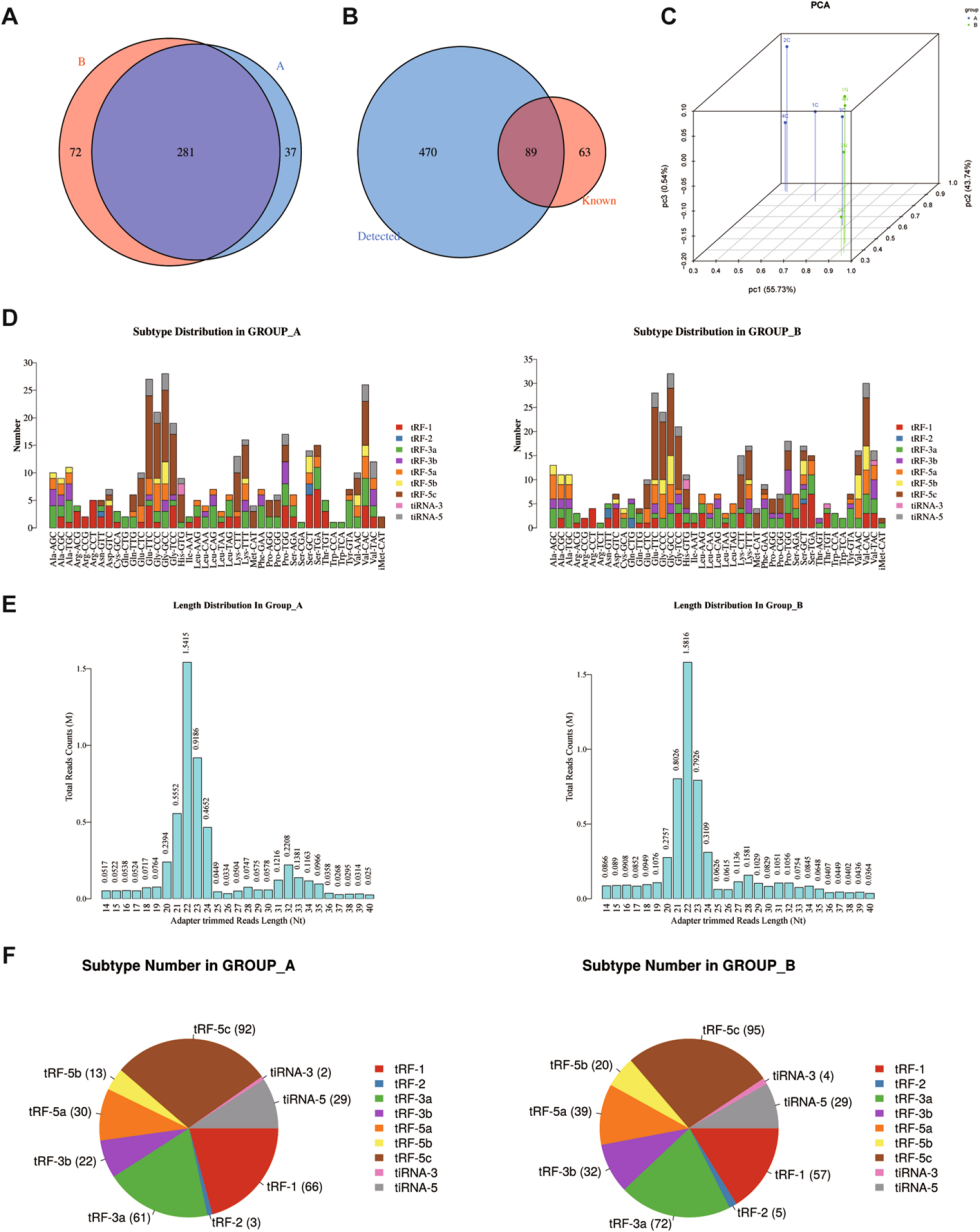
Characteristics of tsRNA profiles in LSCC samples (Group A for non-tumor adjacent tissues, Group B for LSCC tissues). **A** Venn diagrams showed that 281 tsRNAs were co-expressed in both groups. **B** The Venn diagram shows the overlap number of tsRNAs found in the sequencing data and tRFdb. **C** Principal component analysis (PCA) showed a clear difference between groups A and B. **D** Stacked plots indicated the number of subtypes of tsRNAs derived from the same anti-codon tRNA in the two groups. **E** The bar chart of sequence read length distribution revealed the read counts and the read length for each unique read in LSCC in two groups. **F** Pie charts show the subtype number of tsRNAs in the two groups

To identify and characterise the differentially expressed tsRNAs in LSCC, we elucidated tsRNA expression profiles in four pairs of LSCC and matched non-tumor adjacent samples using high-throughput sequencing technology. The tsRNA sequencing data detected a total of 470 tsRNAs. Venn diagrams showed that 281 tsRNAs were co-expressed in both groups, and 89 tsRNAs were found in the sequencing data and verified by tRFdb (a database for transfer RNA fragments) (Fig. 1A, B). Principal component analysis (PCA) showed a clear difference between LSCC and matched non-tumor adjacent tissues (Fig. 1C). Stacked plots indicated the number of tsRNA subtypes derived from the same anti-codon tRNA in each subgroup (Fig. 1D). The bar chart of the sequence read length distribution revealed the read counts and read length for each unique read in each subgroup (Fig. 1E). Pie charts showed the subtype number of tsRNA and indicated that the tRF-3a subclass was the most differentially abundant major type in the two groups (Fig. 1F).

### ***tRF***^***Tyr***^*** was upregulated in LSCC***

**Fig. 2:**
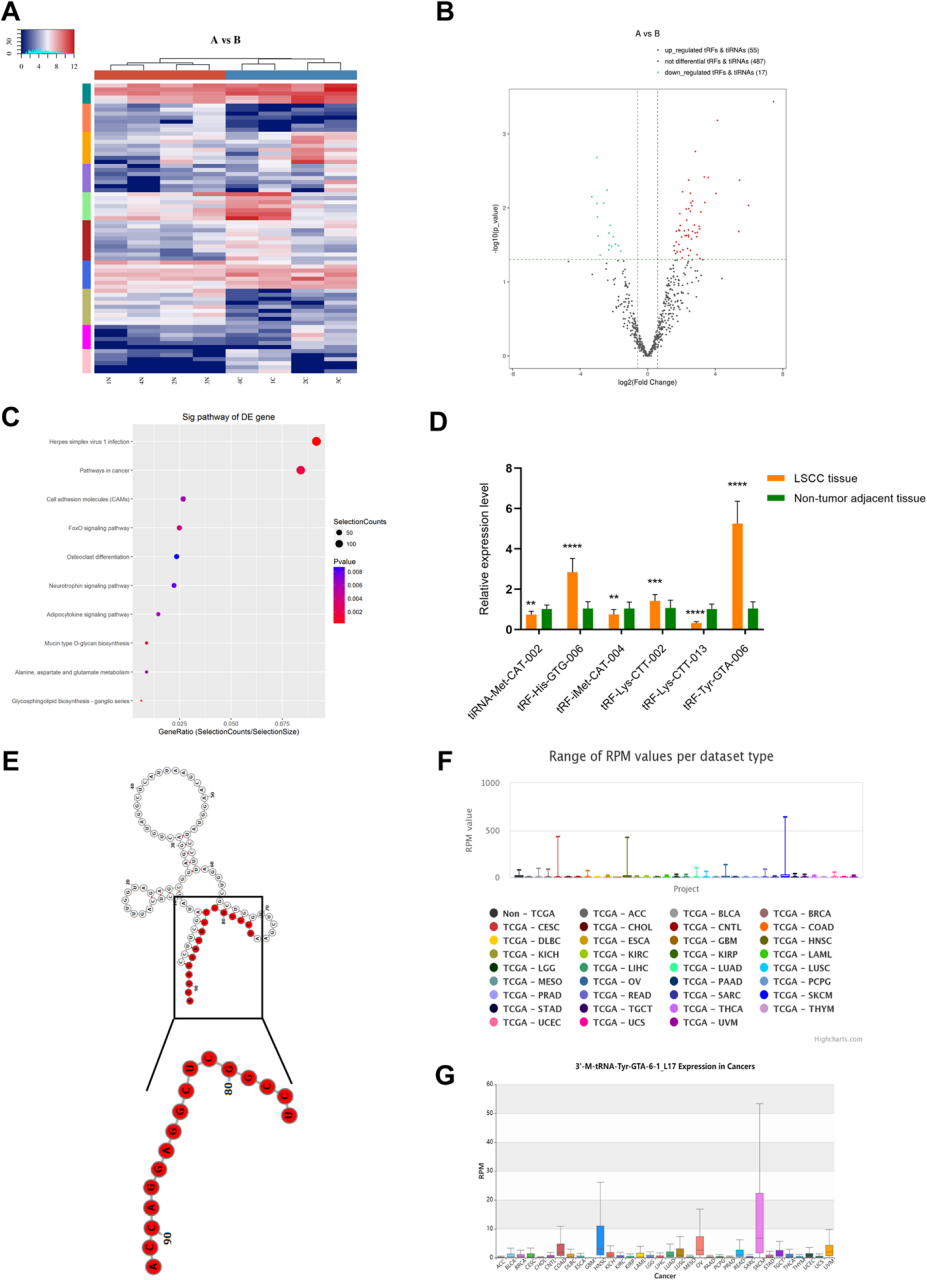
Verification of tsRNA sequencing data and identification of tRF^tyr^ overexpression in LSCC samples. Hierarchical clustering (**A**) and volcano plots (**B**) of the expression profile of tsRNAs. **C** The top ten significant pathways identified by KEGG pathway analysis for differentially expressed tsRNA genes. **D** Verification of the differentially expressed transcripts by qRT–PCR in 60 paired LSCC tissues. The structure of tRF^Tyr^ (**E**) and tRF^Tyr^ expression in various cancers is based on MINTBase (**F**) and OncotRF Database (**G**). *P < 0.05, **P < 0.01, ***P < 0.001, ****P < 0.0001, two-tailed paired *t*-test

With a cut-off criteria of fold change ≥ 1.5 and *P* < 0.05, 72 tsRNAs (including 55 upregulated and 17 downregulated) were identified as being differentially expressed and were visualized by hierarchical clustering analysis and a volcano map (Fig. 2A, B). Differentially expressed genes (DEGs) were enriched in tumour-associated pathways, such as pathways in cancer (Fig. 2C). Three upregulated and three downregulated tsRNAs were chosen to confirm and validate the sequencing data in 60 paired LSCC samples by qRT–PCR (Fig. 2D). We selected the most significantly upregulated tRF^Tyr^ (tRF-17-88481D2, a type of tRF-3a) from 60 pairs of LSCC samples for further analysis. The structure and expression of tRF^Tyr^ were analysed using the oncotRF database and MINTBase (Fig. 2E). The data also showed that tRF^Tyr^ expression was upregulated in head and neck squamous carcinoma (Fig. 2F, G). For LSCC, overexpression of tRF^Tyr^ had a notable correlation with T classification (*P* = 0.0195), lymph node metastasis (*P* = 0.0350), advanced TNM clinical stage (*P* = 0.0371) (Additional file 1: Table S6) and poor prognosis (Additional file 1: Fig. S3).

### tRF^Tyr^-mediated proliferation and metastasis of LSCC

**Fig. 3:**
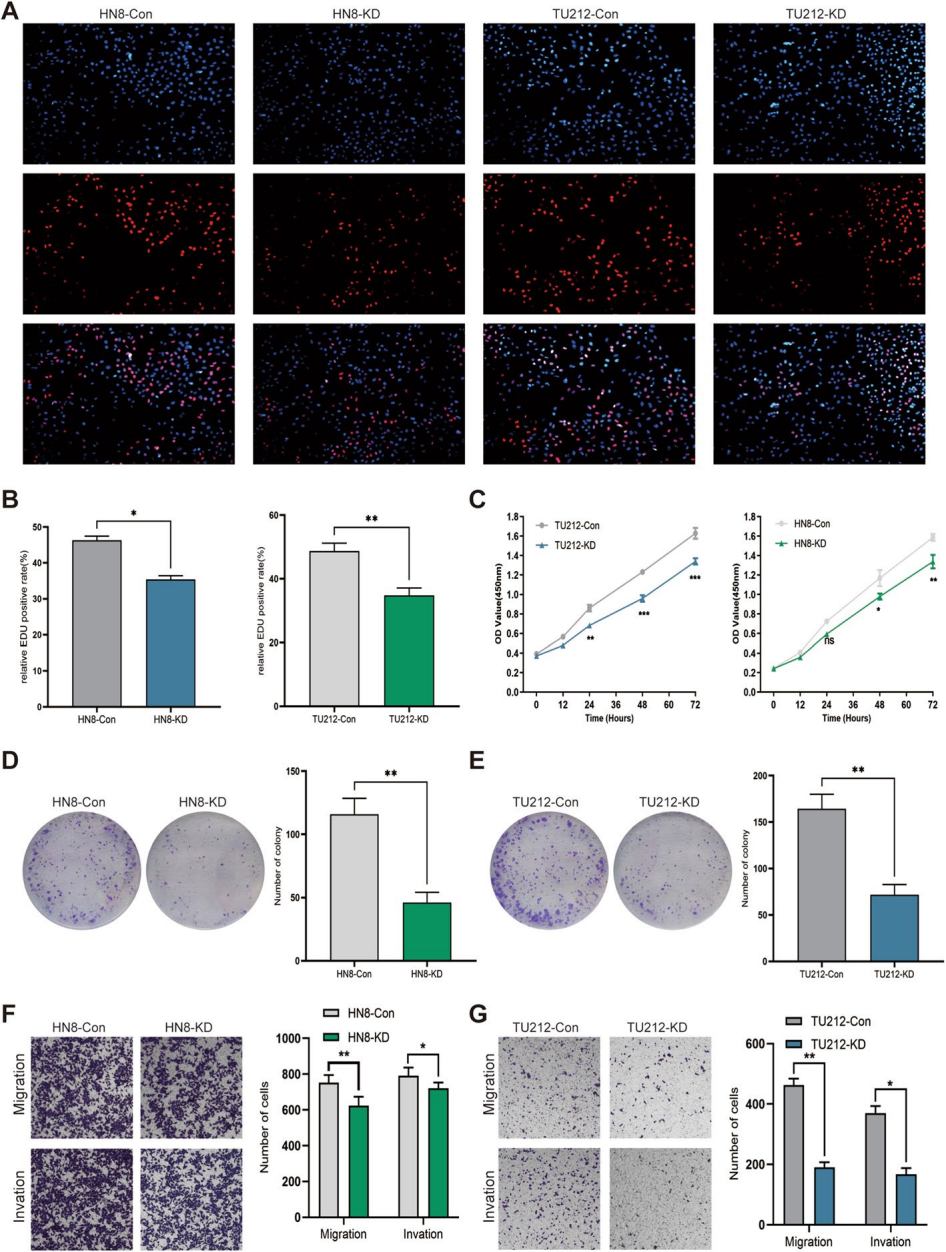
tRF^tyr^-mediated modulation of LSCC progression in vitro. **A** DNA synthesis monitored by EdU labelling of the indicated laryngeal cancer cells (tRF^Tyr^-KD and tRF^Tyr^-Con). The red fluorescence signal indicates EdU-positive cells, and the blue fluorescence signal indicates nuclei. **B** The average relative EdU-positive cell rate was calculated to indicate laryngeal cancer cells. **C** Growth curves of tRF^Tyr^-KD and tRF^tyr^-Con cells were generated by a CCK-8 assay. **D**, **E** A representative colony formation assay in the indicated LSCC cells was conducted. **F**, **G** Transwell assays were conducted to detect the migratory and invasive abilities of the indicated laryngeal cancer cells

The expression of tRF^Tyr^ was detected in various LSCC cell lines, including AMC-HN8, TU212 and TU686 (Additional file 1: Fig. S4A). According to the qRT–PCR data, AMC-HN8 and TU212 cells with relatively high tRF^Tyr^ expression were selected for loss-of-function analysis of tRF^Tyr^. We transfected the Lenti-shRNA vector system (GV280-EGFP) for tRF^Tyr^ expression into cells, and the relative expression data of tRF^Tyr^ are shown in Additional file 1: Fig. S4B–C. EdU, CCK-8 and colony formation assays indicated that tRF^Tyr^ downregulation markedly inhibited tumour cell growth and colony formation in laryngeal cells (Fig. 3A–E). Transwell assays showed that tRF^Tyr^ knockdown suppressed the migration and invasion abilities of LSCC cells (Fig. 3F, G).

### tRF^Tyr^ promotes lactate accumulation in LSCC by binding LDHA

**Fig. 4:**
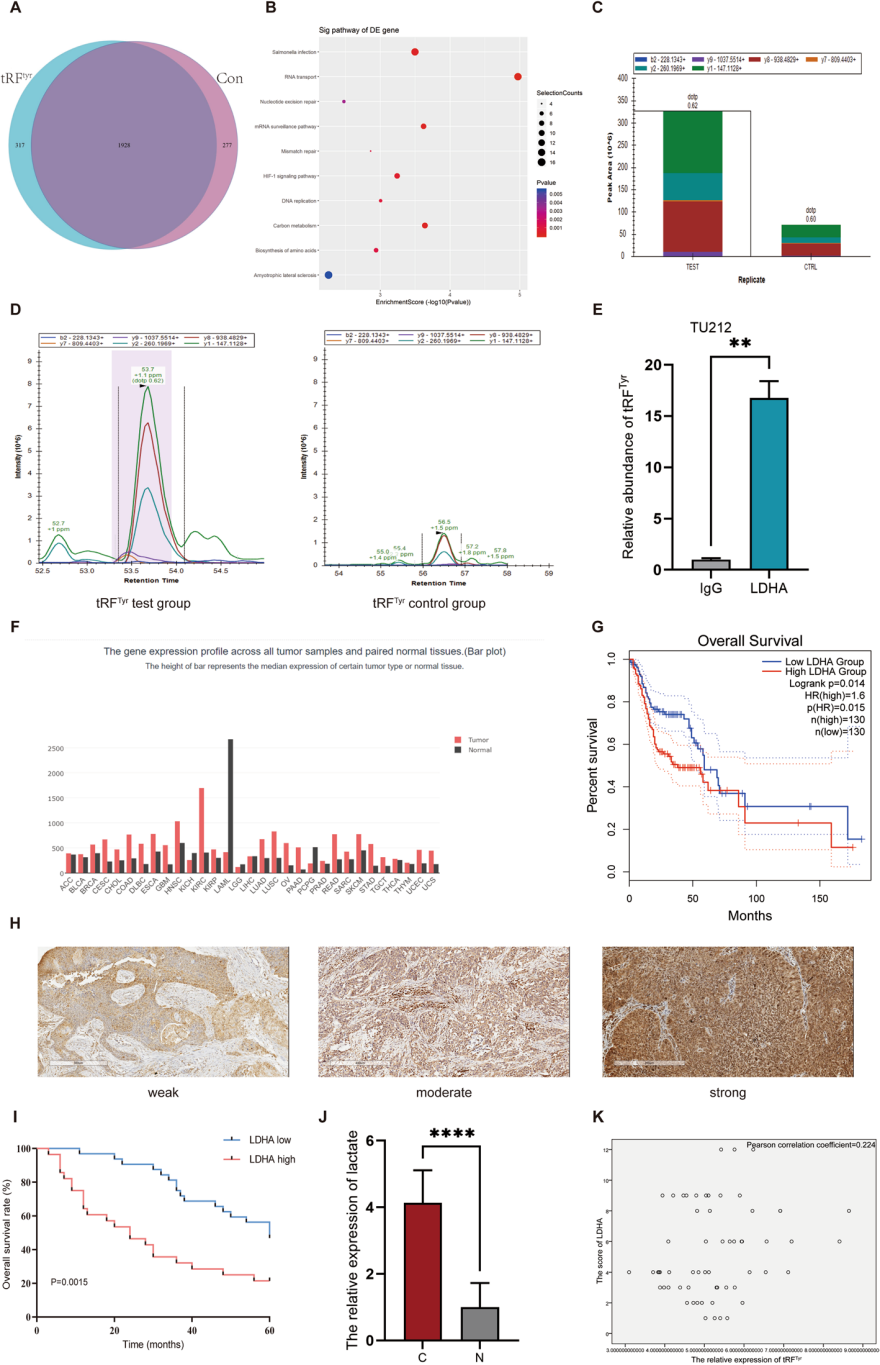
tRF^Tyr^ could specifically bind to LDHA in LSCC. **A** The Venn diagram indicates the number of tsRNAs in tRF^Tyr^ pulldown analysis. **B** The top ten pathways of 317 tRF^Tyr^-specific binding proteins. **C** Histograms of quantitative analysis of LDHA peptides in each subgroup of PRM analysis. **D** Qualitative peak spectrum analysis for the LDHA peptides. **E** RIP assay demonstrated that tRF^Tyr^ was abundantly enriched in the LDHA group compared with the IgG group. **F** The expression of LDHA in various oncologic tissues compared with adjacent normal tissue based on GEPIA. **G** The overall survival of LDHA in HNSC based on GEPIA. **H** Representative immunohistochemical staining of LDHA at different intensities in 60 LSCC tissues. **I** Kaplan–Meier overall survival analysis of LDHA expression in 60 pairs of laryngeal carcinoma tissues. **J** The relative expression of lactate in LSCC samples by LC–MS (C: LSCC tissues, N: adjacent normal tissues). **K** Pearson correlation analysis indicated the relation between tRF^Tyr^ and LDHA expression

**Fig. 5:**
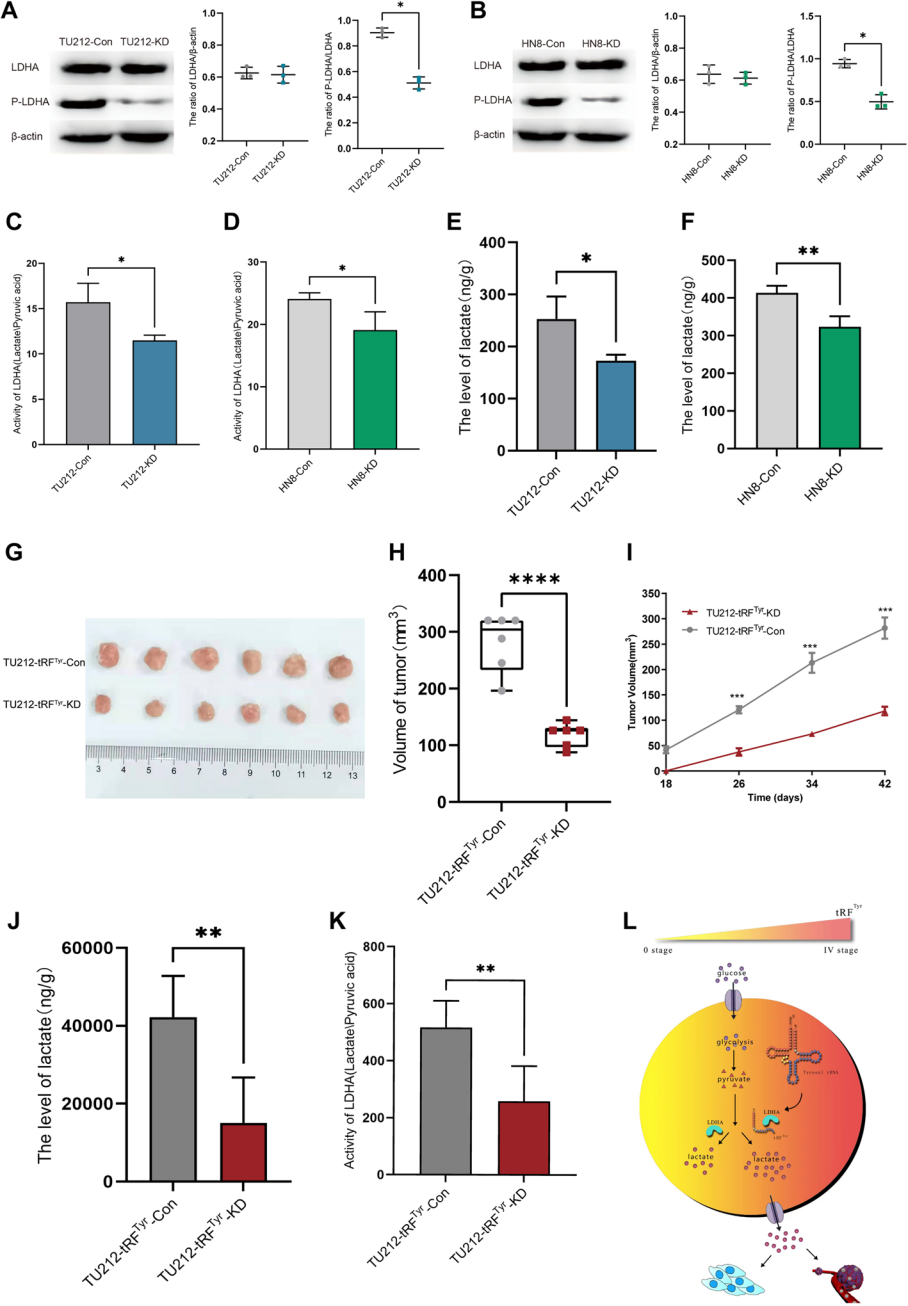
The level of lactate and the activity of LDHA were attenuated after tRF^Tyr^ knockdown. **A**, **B** The expression of LDHA in laryngeal cancer cells by western blot analysis. **C**, **D** The activity of LDHA (lactate/pyruvic acid) in the indicated cell lines. **E**, **F** The level of lactate in the indicated cell lines. **G**–**I** The xenograft tumours of the TU212-tRF^Tyr^-KD group and TU212-tRF^Tyr^-Con group, including the growth curve and tumour volume (*n* = 6/group). **J**–**K** The level of lactate and the activity of LDHA in each indicated xenograft group. **L** Schematic diagram showing that tRF^Tyr^ enhances lactate accumulation and promotes the progression of LSCC by binding LDHA

To explore the mechanism by which tRF^Tyr^ affects laryngeal cancer progression, we used tRF^Tyr^ pull-down analysis to screen for tRF^Tyr^ binding proteins. The LC–MS total ion chromatogram (TIC) is shown in Additional file 1: Fig. S5. The data showed that 317 proteins could specifically bind to tRF^Tyr^; these proteins were significantly enriched in cancer-associated pathways, such as carbon metabolism or the HIF-1 pathway (Fig. 4A, B). We selected the relevant proteins for further verification using PRM assays (Fig. 4C, D). LDHA has been chosen for RIP assay (Fig. 4E). The data indicated that tRF^Tyr^ could specifically bind with LDHA. LDHA is a key enzyme in the conversion of pyruvate to lactate during glycolysis. Based on GEPIA (a web server for gene expression profiling analysis, http://gepia.cancer-pku.cn/), LDHA was overexpressed in HNSC tissues compared with adjacent normal tissues and was notably associated with a poor HNSC prognosis (Fig. 4F, G). In addition, we detected the expression level of LDHA and its direct product lactate in LSCC samples by immunohistochemistry and LC‒MS. The data showed that LDHA was overexpressed and has significant correlation with the prognosis in LSCC (Fig. 4H, I). The level of lactate was also upregulated in LSCC samples (Fig. 4J). Both the LDHA and lactate expression had statistical significance with the clinical parameters of LSCC (Additional file 1: Tables S7, S8). However, Pearson correlation analysis indicated that there was no significant correlation between tRF^Tyr^ and LDHA expression (Fig. 4K). Western blot analysis also verified that LDHA expression was not changed after knockdown of tRF^Tyr^. However, the phosphorylation level of LDHA was significantly decreased after knocking down tRF^Tyr^ (Fig. 5A, B). We then measured the levels of lactate and pyruvate in tRF^Tyr^-mediated LSCC cells using LC–MS analysis. The standard curves of lactate and pyruvic acid are displayed in Additional file 1: Fig. S6. Surprisingly, we found that LDHA activity (the ratio of lactate to pyruvate) was altered by tRF^Tyr^. Both the level of lactate and the activity of LDHA were significantly decreased after tRF^Tyr^ knockdown in vitro (Fig. 5C–F). For further verification in vivo, we constructed a xenograft model in nude mice by injecting cells stably transfected with tRF^Tyr^-shRNA lentivirus (*n* = 6/group). As illustrated in Fig. 5G–I, the volume of xenografts and tumour growth rate in the tRF^Tyr^ knockdown group were significantly reduced compared with those in the control group. We then measured the levels of lactate and pyruvate in vivo. Accordingly, the data revealed that the level of lactate and the activity of LDHA were significantly decreased after tRF^Tyr^ knockdown in xenografts (Fig. 5J, K). Thus, our research indicated that tRF^Tyr^ could enhance lactate accumulation and promote the progression of LSCC by binding with LDHA (Fig. 5L).

## Discussion

Laryngeal cancer is a complex disease with multiple dysregulated gene loci [27]. However, its pathogenesis remains elusive. With the improvement of therapeutic concepts and approaches, most patients with early stage laryngeal cancer can retain laryngeal function after treatment. Unfortunately, lymph node metastasis and tumour recurrence are the main causes of poor prognosis in LSCC. Hence, there is an urgent need to identify more biomarkers to improve the early diagnosis and treatment of laryngeal cancer.

With the development of next-generation sequencing, tsRNAs derived from tRNAs have been identified as a new type of small non-coding RNA with multiple modifications [28]. tsRNAs represent a new generation of clinical biomarkers in view of their high stability and specificity. An increasing number of studies in recent years have confirmed that tsRNAs are dysregulated in various physiological cellular processes and diseases, especially in tumours [29]. Recently, an overview in the New England Journal of Medicine confirmed that tsRNAs could play an important regulatory role as biomarkers and therapeutic targets in tumours. As reported, LeuCAG3tsRNA, a tRNA-derived small RNA (tsRNA), could promote cell growth and proliferation in liver tumours via the translation of two mRNAs (RPS28 and RPS15), which can be transcribed into ribosomal proteins [8, 30]. However, its role and mechanism in laryngeal cancer remain unclear.

We delineated the expression landscape of tsRNAs in four paired LSCC samples by tsRNA sequencing. The data analysis identified 72 dysregulated tsRNAs in LSCC tissues. We then selected several aberrant genes to verify the sequencing data by qRT–PCR in 60 paired LSCC samples. tRF^Tyr^ was found to be significantly upregulated in LSCC (Fig. 1). Functional assays indicated that tRF^Tyr^ knockdown notably suppressed the malignant phenotype of laryngeal cancer cells (Fig. 3). Mechanistic experiments have shown that tRF^Tyr^ specifically binds to LDHA. LDHA and lactate were significantly upregulated and may be a prognostic biomarker for LSCC (Fig. 4). However, there were no obvious changes in LDHA expression when tRF^Tyr^ was silenced. Surprisingly, the phosphorylation level of LDHA and its activity were significantly attenuated after knockdown of tRF^Tyr^. Accordingly, knockdown of tRF^Tyr^ resulted in reduction of the lactate levels in vitro and in vivo (Fig. 5). The constant proliferation of tumour cells requires a large amount of oxygen consumption. Glycolysis converts glucose into lactate as the main mode of energy supply for tumour cells under hypoxia [31]. The accumulation of lactate in tumour cells could in turn acidify the hypoxic microenvironment to facilitate the progression of cancers [32–34]. Recently, a novel histone modification mediated by lactate reaffirmed the important regulatory role of lactate in cancers and other diseases [35–37]. LDHA, as an important enzyme, converts pyruvate to lactate in aerobic glycolysis [38]. Reportedly, the activity of LDHA could directly affect the level of lactate [39]. Tumour or other hypoxia-related cells change the cell microenvironment and promote cell growth by regulating the activity of LDHA [39–41]. LDHA activity can be changed by many factors [42–44]. The phosphorylation level of LDHA could directly activate LDHA and enhance its bioactivity in various diseases, including tumours [45]. For example, LDHA was reported to interact with upstream molecules (kinase HER2 and Src) to phosphorylate LDHA at Y10 and enhance its activity, thus promoting the progression of metastatic breast cancer [45]. In addition, tsRNA could help tumour cells adapt to the hypoxic environment for the rapid growth of tumours [46]. The Journal of Science has published a new mechanistic mechanism of non-coding RNA, which can directly bind to the signal molecule STAT3 and protect its phosphorylated site to regulate immune cells [47]. Similarly, our data demonstrated that tRF^Tyr^ could interact with LDHA and influence its phosphorylation to promote the progression of LSCC, which may be a novel functional mode of the tsRNA mechanism.

## Conclusions

This study identified that tRF^Tyr^, as a novel oncogene, could induce lactate accumulation and promote the tumour progression of LSCC by binding to LDHA. Our data provide new insights for the exploration of tsRNAs and highlight the role of tRF^Tyr^ in LSCC, which may become a new therapeutic target for cancer treatment.

## Supplementary Information

**Additional file 1: Figure S1.** tRF & tiRNA-seq quality score plot. **Figure S2.** Representative raw real-time PCR and western blot data. **Figure S3.** Kaplan–Meier overall survival analysis of tRF^Tyr^ expression in 60 pairs of laryngeal carcinoma tissues. **Figure S4.** The transfection of tRF^Tyr^ in LSCC cell lines. **Figure S5.** The LC–MS total ion chromatogram from RNA pull-down. **Figure S6.** The standard curve of lactateand Pyruvic acid. **Table S1.** RNA quantification and quality assurance by NanoDrop ND-1000. **Table S2.** Quality score.** Table S3.** Mapping summary. **Table S4.** The details of the selected tRF transcripts. **Table S5.** The sequences of shRNA. **Table S6.** Relationship between tRF^tyr^ expression and clinicopathological features of LSCC. **Table S7.** Relationship between LDHA expression and clinicopathological features of LSCC. **Table S8.** Relationship between the level of lactate and clinicopathological features of LSCC. **Method S1.** Immunohistochemistry. **Method S2.** Western blot analysis.

## Data Availability

The data supporting the findings of this study are available from the corresponding author upon reasonable request.

## Abbreviations

ESI: Electrospray ionization
GTEx: Genotype tissue expression
HNSC: Head and neck squamous cell carcinoma
GEPIA2: Gene expression profiling interactive analysis
IHC: Immunohistochemical
LDHA: Lactate dehydrogenase A
LC–MS: Liquid chromatography–mass spectrometry
mRNA: Messenger RNA
tiRNA-5: 5′-tRNA halves
MRM: Multiple response monitoring
CCK-8: Cell counting kit 8
tiRNA-3: 3′-tRNA halves
qRT-PCR: Quantitative real-time PCR
PRM: Parallel reaction monitoring
sncRNA: Small non-coding RNA
LSCC: Laryngeal squamous cell carcinoma
tsRNA: Transfer RNA-derived small RNA
tRNAs: Transfer RNAs
TCGA: The Cancer Genome Atlas
