# Supplementary material for: A novel tyrosine tRNA-derived fragment, tRFTyr, induces oncogenesis and lactate accumulation in LSCC by interacting with LDHA
Source: Cell Mol Biol Lett. 2023 Jun 26;28:49. doi: 10.1186/s11658-023-00463-8 (PMC10291753; doi:10.1186/s11658-023-00463-8)
Supplement: Supplementary file 1 — Additional file 1: Figure S1. tRF & tiRNA-seq quality score plot. Figure S2. Representative raw real-time PCR and western blot data. Figure S3. Kaplan–Meier overall survival analysis of tRFTyr expression in 60 pairs of laryngeal carcinoma tissues. Figure S4. The transfection of tRFTyr in LSCC cell lines. Figure S5. The LC–MS total ion chromatogram from RNA pull-down. Figure S6. The standard curve of lactateand Pyruvic acid. Table S1. RNA quantification and quality assurance by NanoDrop ND-1000. Table S2. Quality score. Table S3. Mapping summary. Table S4. The details of the selected tRF transcripts. Table S5. The sequences of shRNA. Table S6. Relationship between tRFtyr expression and clinicopathological features of LSCC. Table S7. Relationship between LDHA expression and clinicopathological features of LSCC. Table S8. Relationship between the level of lactate and clinicopathological features of LSCC. Method S1. Immunohistochemistry. Method S2. Western blot analysis. [file 11658_2023_463_MOESM1_ESM.doc]

**Additional file 1**

**Additional figs and fig legends:**

**Figure S1:**

**
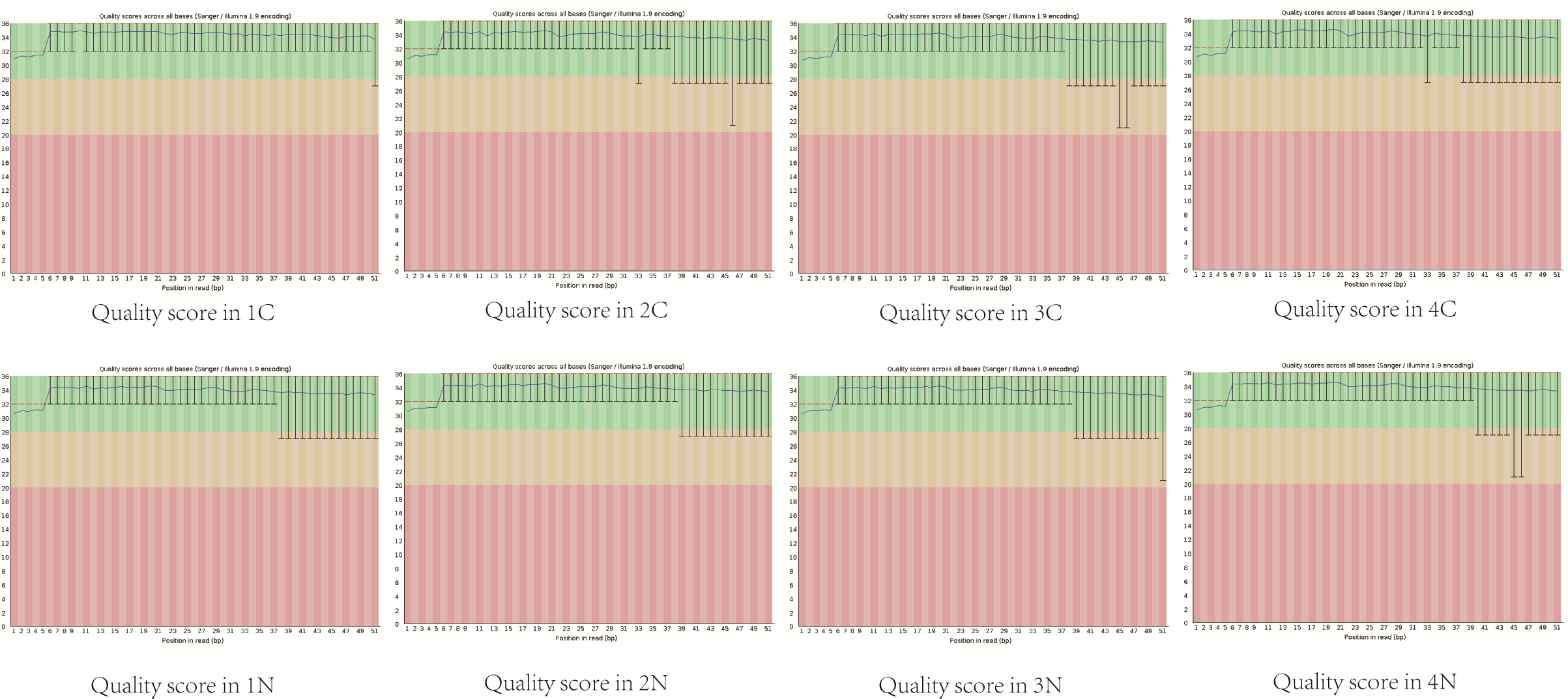
**

**Fig. S1 tRF & tiRNA-seq quality score plot.** The position in the read is plotted on the X-axis and the Q value is plotted on the Y-axis. The red line is the median Q score, and the blue line is the mean Q score. The boxplot represents the interquartile range, while the whiskers represent the 10% and 90% points. A Q score above 30 (>99.9% correct) is considered high-quality data.

**Figure S2A:**


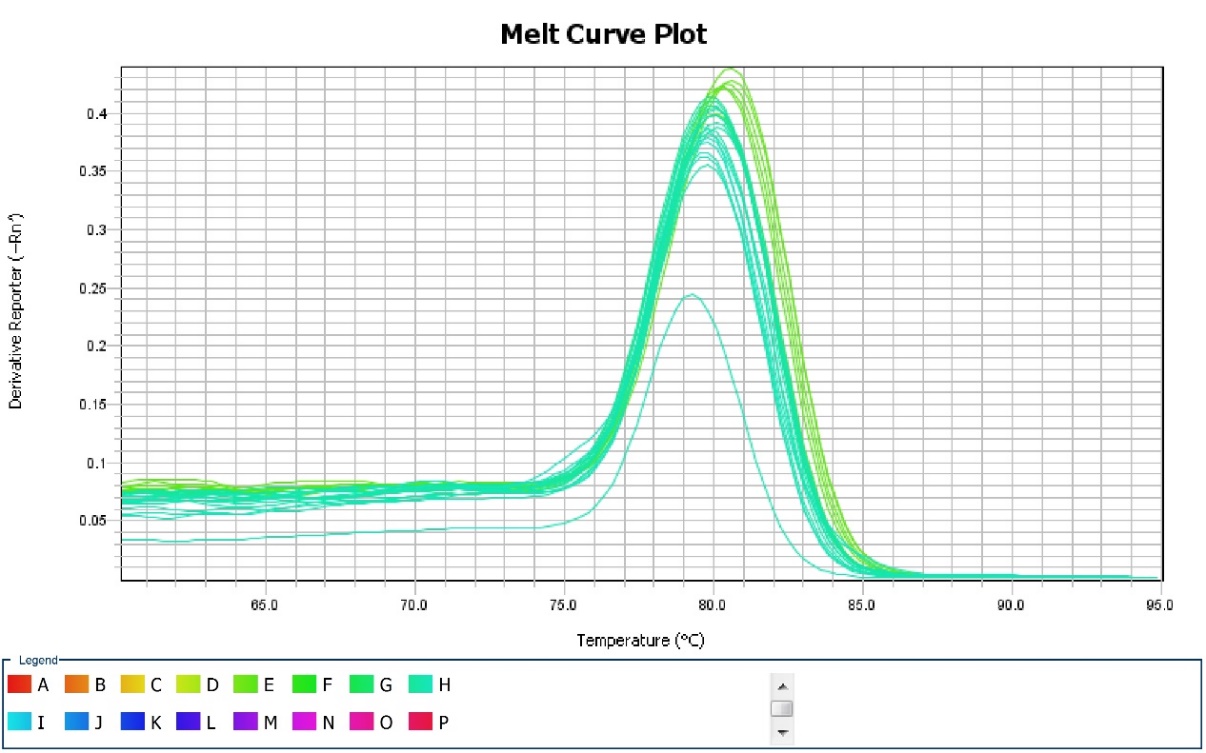


Melt Curve Plot of tRF-tyr-GTA-006


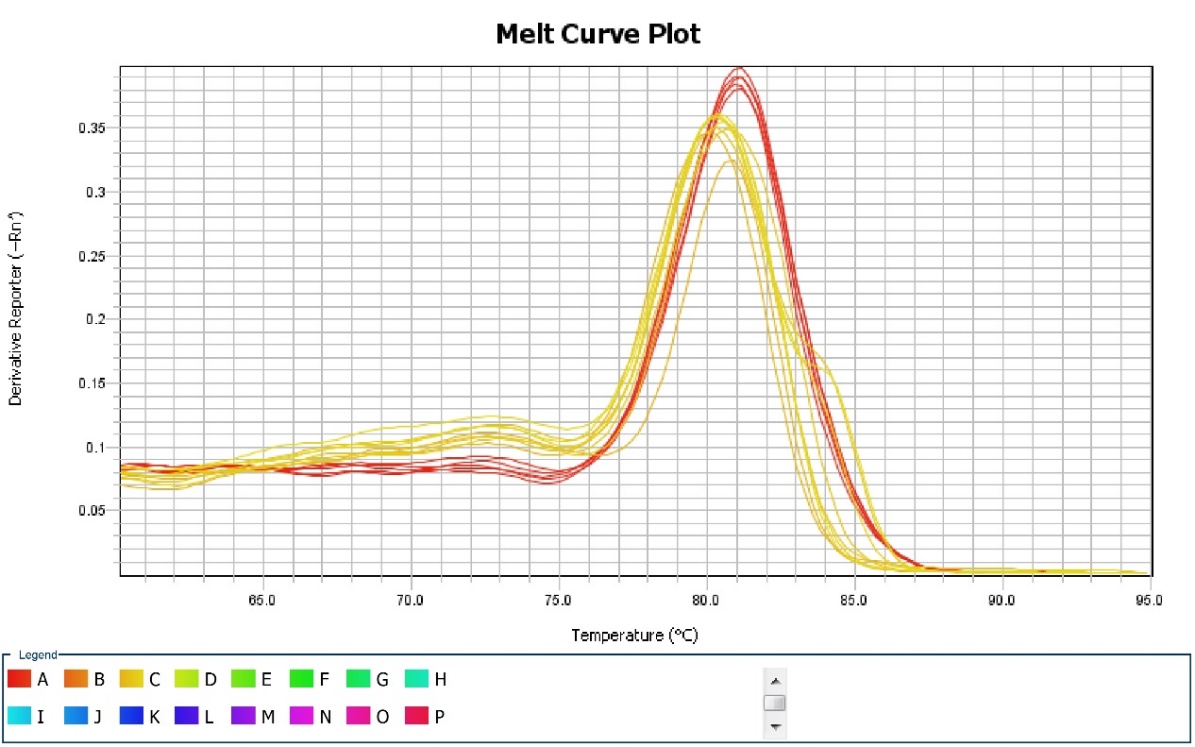


Melt Curve Plot of tRF-Lys-CTT-013


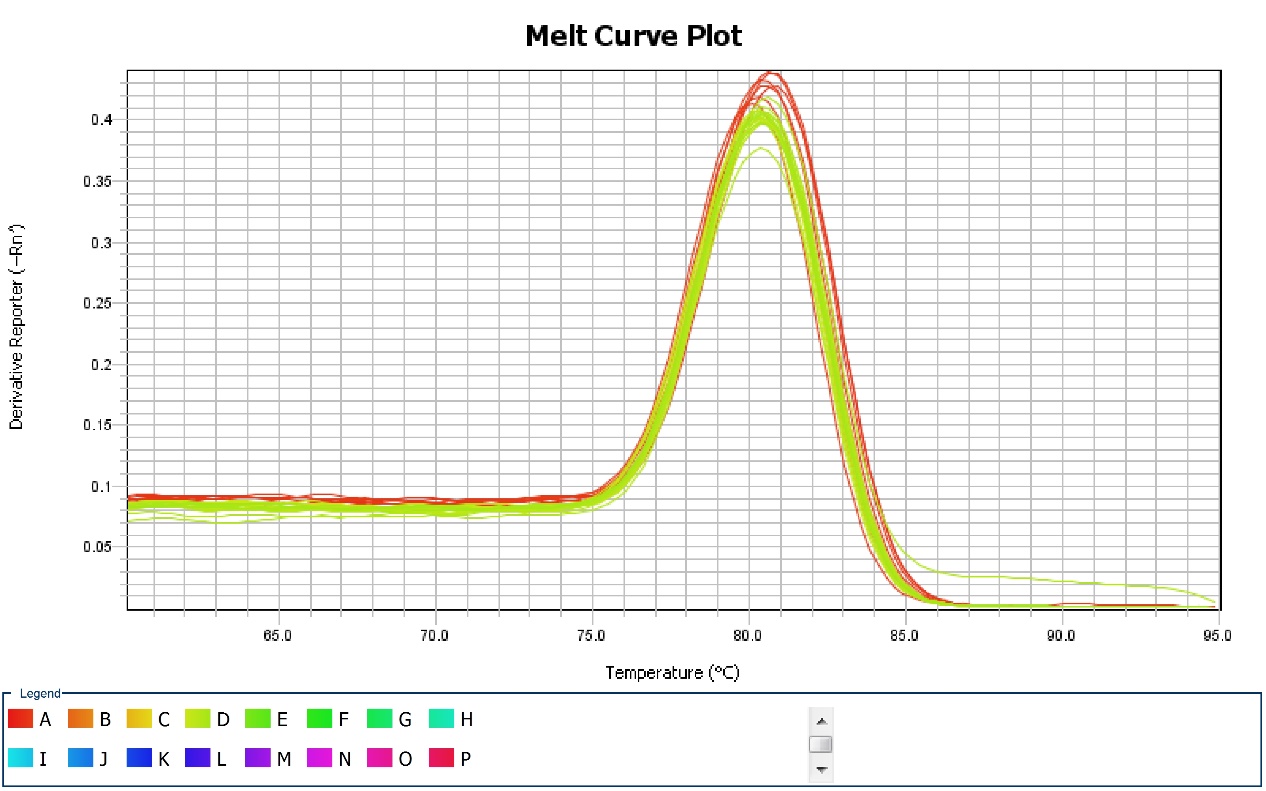


Melt Curve Plot of tRF-Lsy-CTT-002


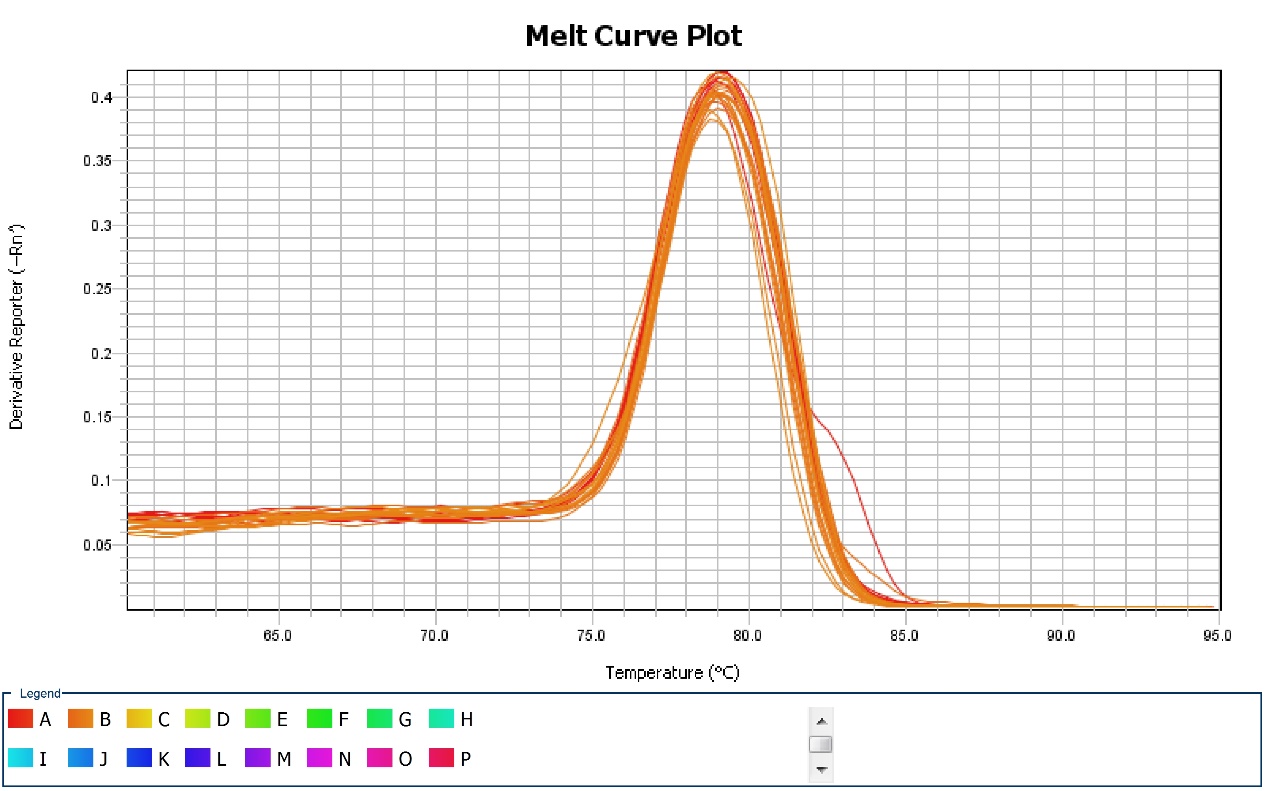


Melt Curve Plot of tRF-iMet-CAT-004


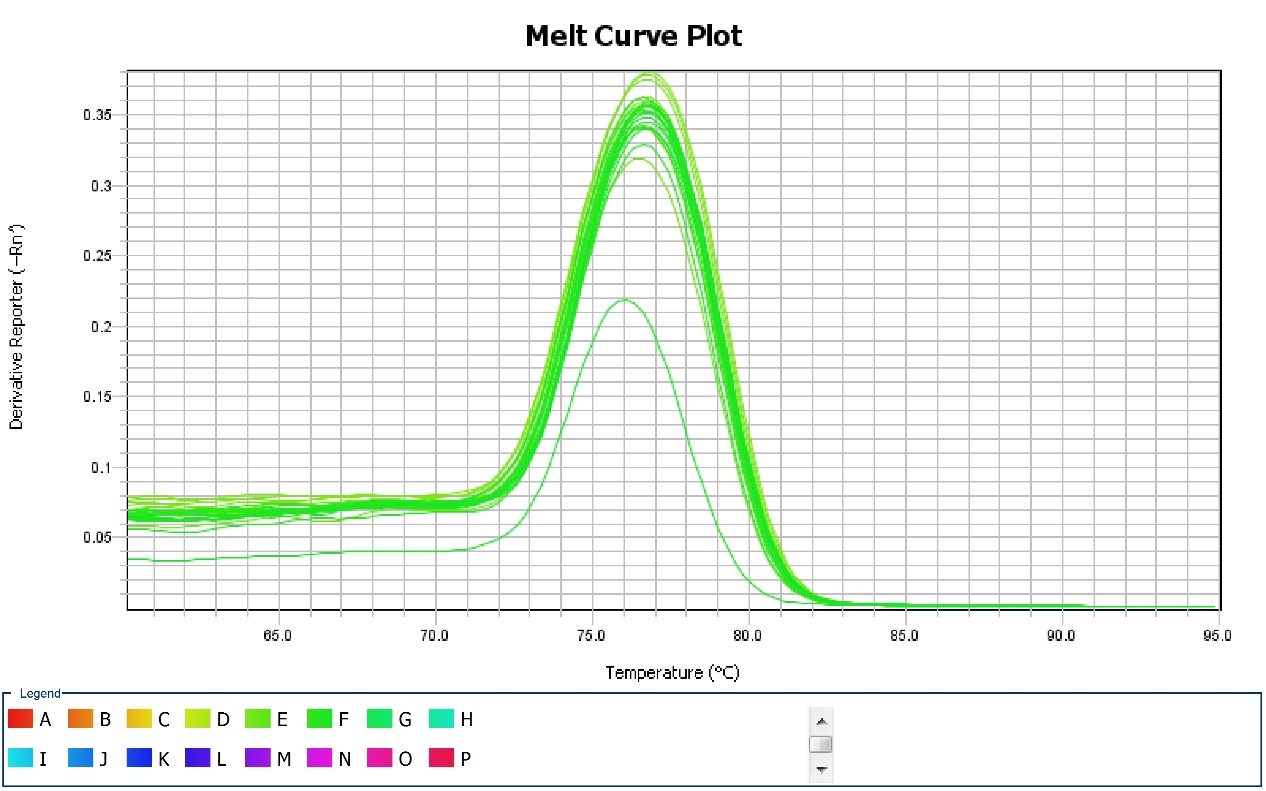


Melt Curve Plot of tRF-His-GTG-006


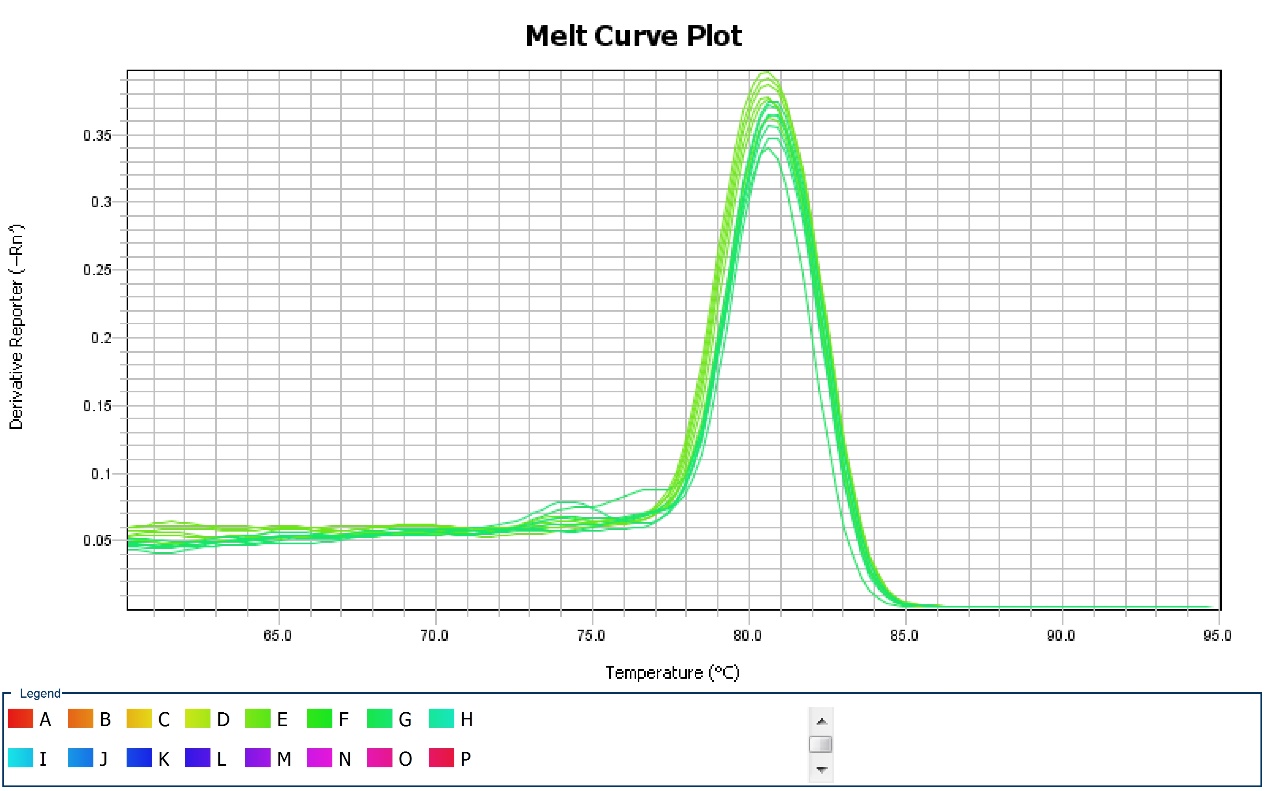


Melt Curve Plot of tiRNA-Met-CAT-002


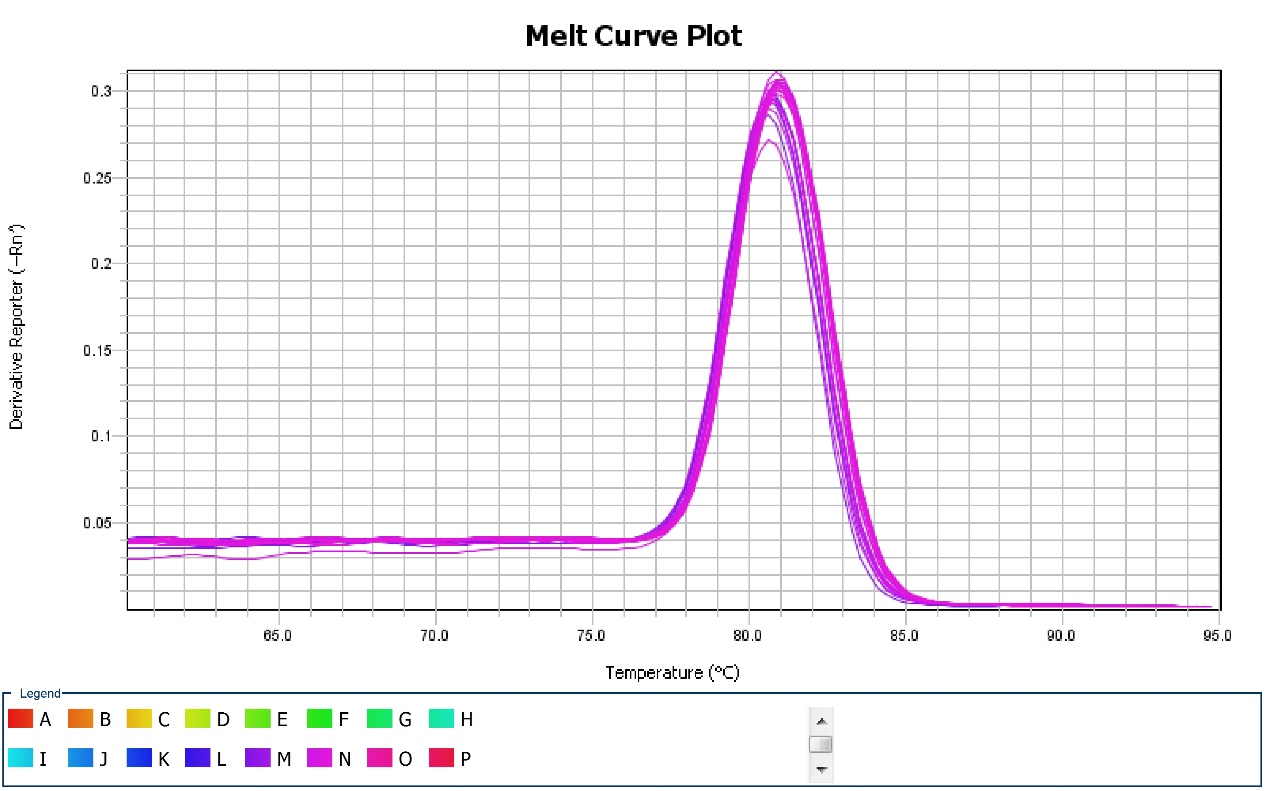


Melt Curve Plot of U6

**Figure S2B:**

**
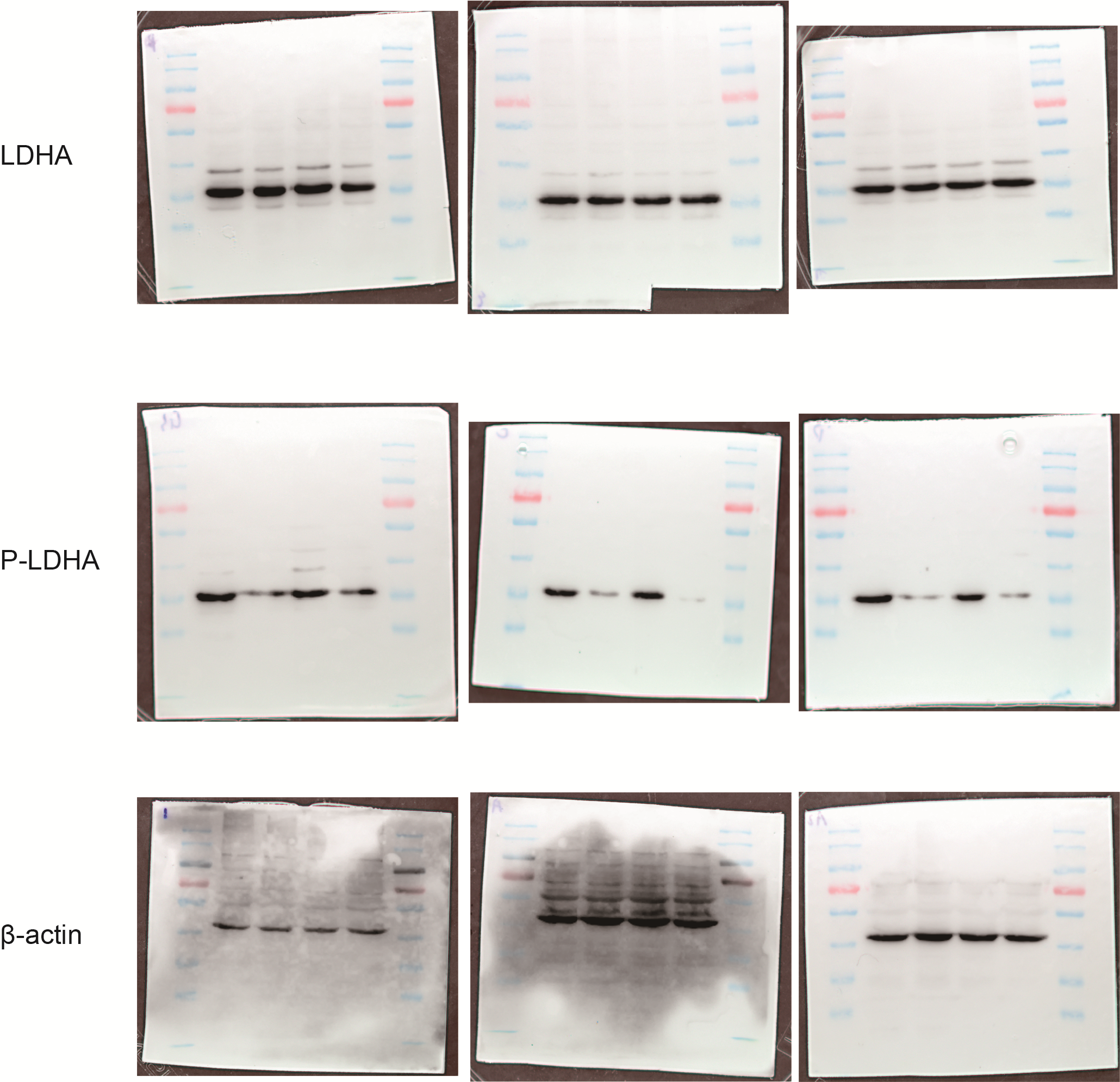
**

**Fig. S2 Representative raw real-time PCR and western blot data. A)** Melt curve of tsRNAs. **B)** The raw films of the western blot in triplicate.

**Figure S3**

**
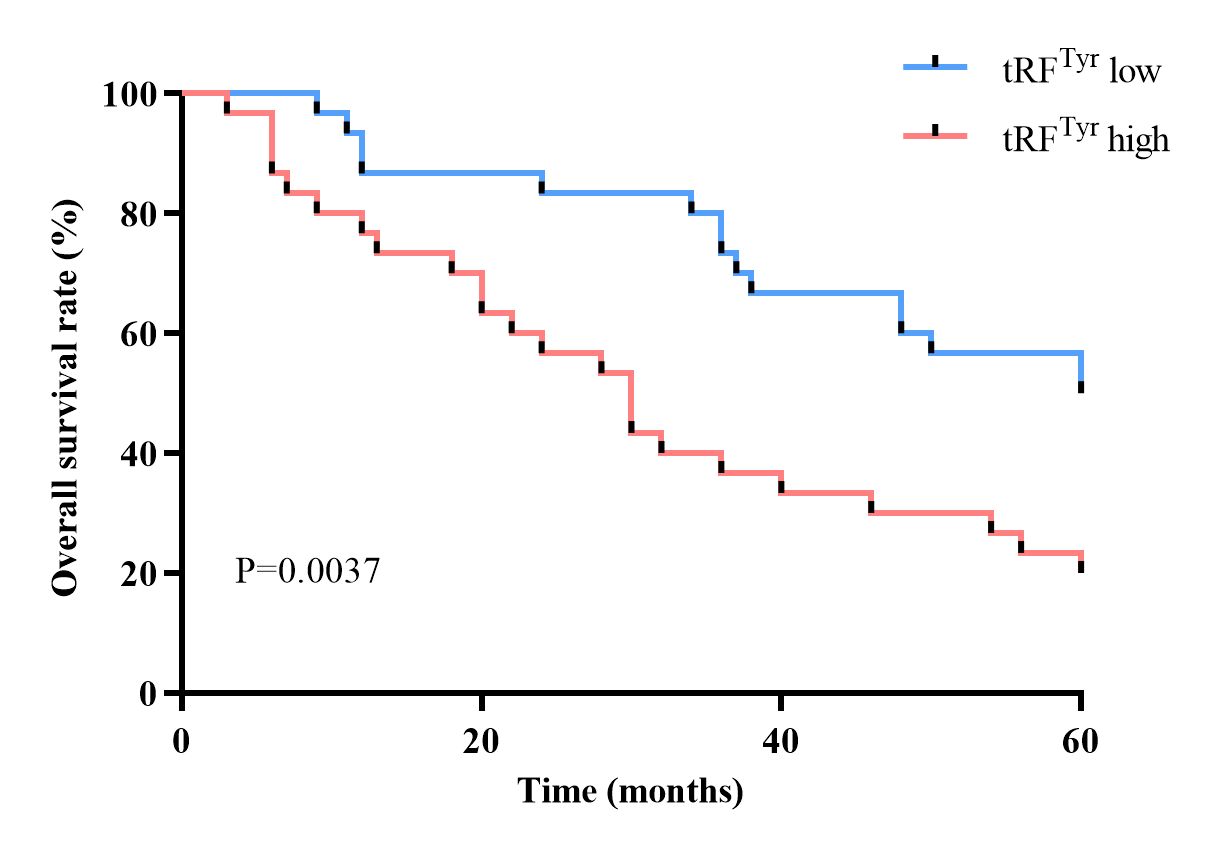
**

**Fig. S3 Kaplan–Meier overall survival analysis of tRF^Tyr^ expression in 60 pairs of laryngeal carcinoma tissues**.

**Figure S4:**

**
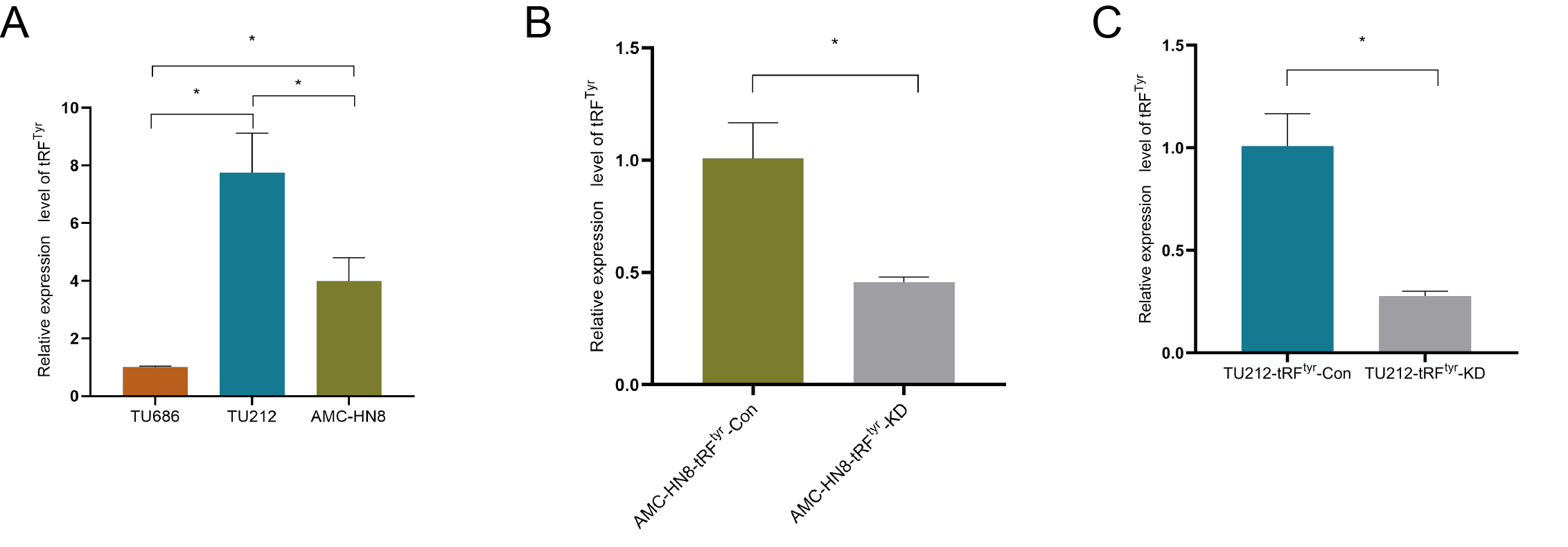
**

**Fig. S4 The transfection of tRF^Tyr^ in LSCC cell lines. A)** The expression level of tRF^Tyr^ in the indicated LSCC cell lines (AMC-HN8, TU212, and TU686). B-C) The relative expression level of tRF^Tyr^ in the cells transfected with the Lenti-shRNA vector and the control group cells.

**Figure S5**

**
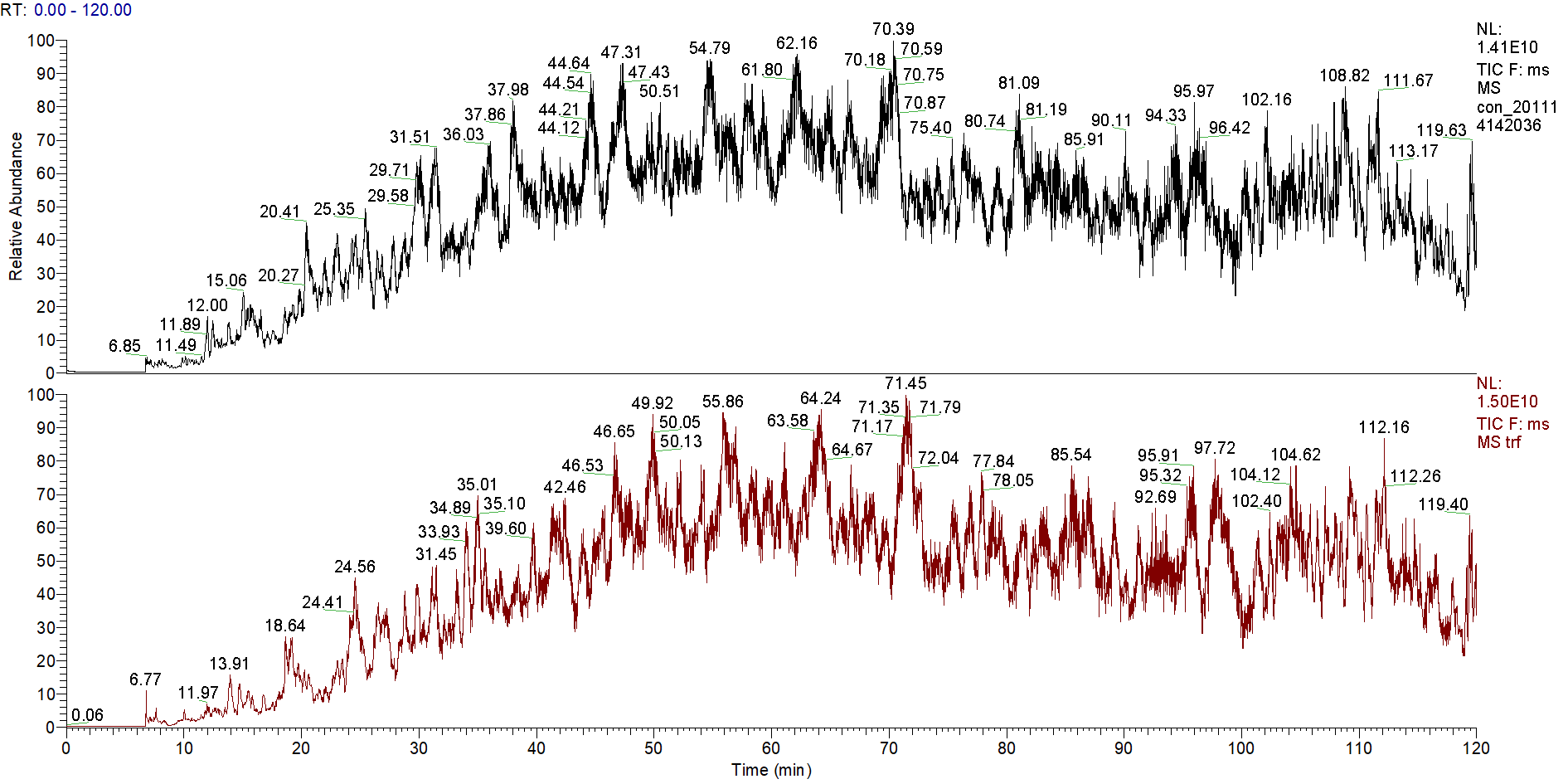
**

**Fig. S5 The LC-MS total ion chromatogram (TIC) from RNA pull-down**

**Figure S6**

**
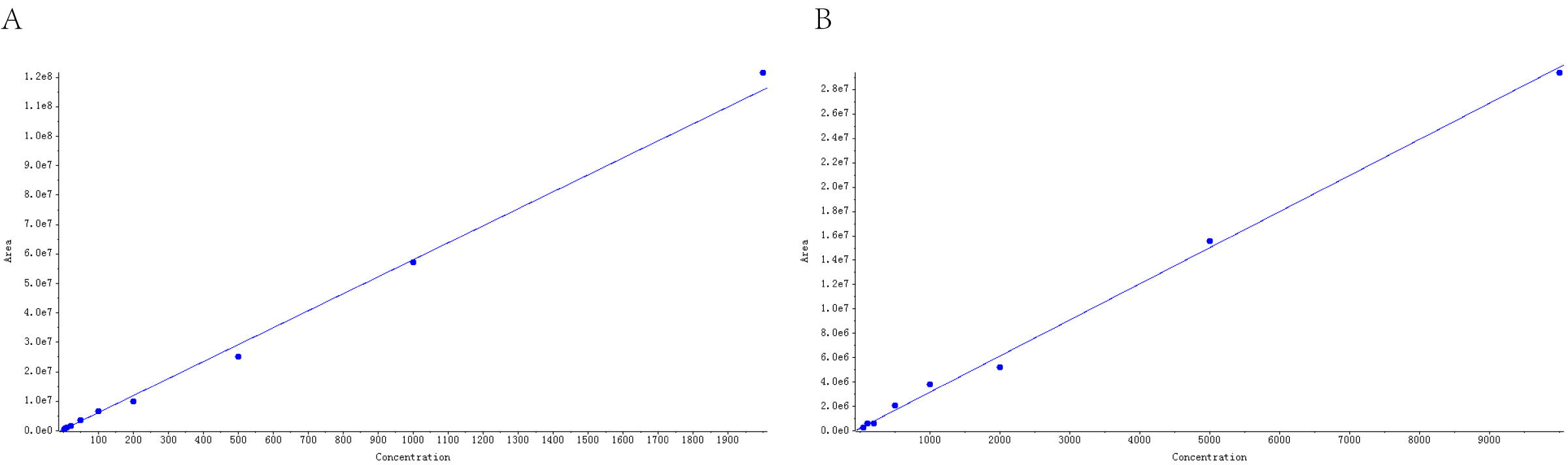
**

**Fig. S6** **The standard curve of lactate (A)and Pyruvic acid (B).**

**Additional tables and table legends:**

**Table S1** **RNA Quantification and Quality Assurance by NanoDrop ND-1000**

| **Sample ID** | **OD260/280**  **Ratio** | **OD260/230**  **Ratio** | **Conc. (ng/μl)** | **Volume (μl)** | **Quantity (ng)** | **QC Purity Pass or Fail** |
| --- | --- | --- | --- | --- | --- | --- |
| 4C | 1.99 | 2.33 | 942.58 | 100 | 94258.00 | Pass |
| 4N | 1.96 | 2.30 | 642.04 | 100 | 64204.00 | Pass |
| 1C | 1.98 | 2.32 | 897.31 | 100 | 89731.00 | Pass |
| 1N | 1.89 | 2.28 | 353.43 | 100 | 35343.00 | Pass |
| 3C | 1.98 | 2.20 | 1044.13 | 100 | 104413.00 | Pass |
| 3N | 1.89 | 2.24 | 416.48 | 100 | 41648.00 | Pass |
| 2C | 1.93 | 2.28 | 1388.04 | 100 | 138804.00 | Pass |
| 2N | 1.89 | 2.03 | 449.42 | 50 | 22471.00 | Pass |

*For the spectrophotometer, the O.D. A260 /A280 ratio should be close to 2.0 for pure RNA (ratios between 1.8 and 2.1 are acceptable). The O.D. A260/A230 ratio should be more than 1.8.

**Table S2 Quality score**

| **Sample** | **TotalRead** | **TotalBase** | **BaseQ30** | **BaseQ30 (%)** |
| --- | --- | --- | --- | --- |
| 1C | 5088532 | 259515132 | 243598113 | 93.87 |
| 2C | 6599557 | 336577407 | 307868044 | 91.47 |
| 1N | 7546924 | 384893124 | 352387905 | 91.55 |
| 2N | 8503750 | 433691250 | 397268272 | 91.60 |
| 3C | 9697980 | 494596980 | 452784687 | 91.55 |
| 4C | 5813359 | 296481309 | 271490775 | 91.57 |
| 3N | 7253631 | 369935181 | 338544652 | 91.51 |
| 4N | 6294633 | 321026283 | 294282078 | 91.67 |

Sample: Sample name

TotalRead: Raw sequencing reads after quality filtering.

TotalBase: Number of bases after quality filtering.

BaseQ30: Number of bases of Q score more than 30 after quality filtering.

BaseQ30 (%): The proportion of bases (Q>30) number after quality filtering.

**Table S3 Mapping summary**

| **Sample** | **Trimmed** | **Mat-**  **tRNA** | **Mat- tRNA （%)** | **Pre- tRNA** | **Pre- tRNA (%)** | **tsRNA** | **tsRNA (%)** |
| --- | --- | --- | --- | --- | --- | --- | --- |
| 1C | 4193986 | 379208 | 9.04 | 28680 | 0.68 | 3016558 | 71.93 |
| 2C | 5532610 | 391421 | 7.07 | 27878 | 0.50 | 3767406 | 68.09 |
| 1N | 5371031 | 373284 | 6.95 | 21270 | 0.40 | 3325779 | 61.92 |
| 2N | 6963022 | 266404 | 3.83 | 32737 | 0.47 | 3969595 | 57.01 |
| 3C | 6764588 | 754765 | 11.16 | 78615 | 1.16 | 4184519 | 61.86 |
| 4C | 4463846 | 874666 | 19.59 | 32908 | 0.74 | 2767621 | 62.00 |
| 3N | 5634885 | 246390 | 4.37 | 34864 | 0.62 | 3176437 | 56.37 |
| 4N | 4192306 | 347929 | 8.30 | 30528 | 0.73 | 2761616 | 65.87 |

Sample: Sample name

Trimmed: Reads number after 5，,3，-adaptor trimmed and discarded reads (length < 14nt or > 40nt).

Mat-tRNA: Reads number aligned to mature tRNA.

Mat-tRNA(%): The proportion of reads number aligning to mature tRNA.

Pre-tRNA: Reads number aligned to precursor tRNA

Pre-tRNA(%): The proportion of reads number aligning to precursor tRNA

tsRNA: Reads number aligned to miRNA

tsRNA(%): The proportion of reads number aligning to miRNA

**Table S4 The details of the selected tRF transcripts**

| **基因名** | **双向引物序列** | **退火温度(℃)** | **产物长度(bp)** |
| --- | --- | --- | --- |
| U6 | F:5’GCTTCGGCAGCACATATACTAAAAT3’  R:5’CGCTTCACGAATTTGCGTGTCAT3’ | 60 | 89 |
| tRF-iMet-CAT-004 | F:5’ TACAGTCCGACGATCAACCAT3'  R:5’ CTCTTCCGATCTTGGTAGCAG3’ | 60 | 45 |
| tRF-Lys-CTT-013 | F:5’ CAGTCCGACGATCCCCCA 3'  R:5’ TCTTCCGATCTTGGCGCC3’ | 60 | 41 |
| tRF-Lys-CTT-002 | F:5’ TCTACAGTCCGACGATCGCC3'  R:5’ CTCTTCCGATCTCTGAGCTAGCC3’ | 60 | 44 |
| tRF-His-GTG-006 | F:5’ ATCGCCGTGATCGTATAGTGG3'  R:5’ TGTGCTCTTCCGATCTCAGAGTA3’ | 60 | 48 |
| tiRNA-Met-CAT-002 | F:5’ AGTTCTACAGTCCGACGATCA3'  R:5’ TGGTAGTACGGGAAGGGTATAA3’ | 60 | 60 |
| tRF-Tyr-GTA-006 | F:5’ TCTACAGTCCGACGATCTCCG3'  R:5’ CTCTTCCGATCTTGGTCCTCC3’ | 60 | 46 |

**Table S5 The sequences of shRNA**

| **NO.** | **5’** | **STEM** | **3’** |
| --- | --- | --- | --- |
| tRF-17-88481D2-inhibition(65245-1)-b | CCGG | TGGTCCTCCGAGCCGGA | TTTTTg |

**Table S6 Relationship between tRF^tyr^ expression and clinicopathological features of LSCC**

|  |  |  | **tRF^tyr^ expression** | |  |
| --- | --- | --- | --- | --- | --- |
| **Parameter** | **Group** | **Total** | **Low** | **High** | **P value** |
| Age | <60 | 33 | 16 | 17 | 0.7952 |
|  | ≥60 | 27 | 14 | 13 |  |
| Gender | Male | 43 | 22 | 21 | 0.7745 |
|  | Female | 17 | 8 | 9 |  |
| T classification | T1/T2 | 33 | 21 | 12 | 0.0195（*） |
|  | T3/T4 | 27 | 9 | 18 |  |
| Lymph nodes | Positive | 24 | 8 | 16 | 0.0350（*） |
|  | Negative | 36 | 22 | 14 |  |
| Clinical stage | Ⅰ,Ⅱ | 26 | 17 | 9 | 0.0371（*） |
|  | Ⅲ,Ⅳ | 34 | 13 | 21 |  |

*P < 0.05, **P < 0.01, ***P < 0.001, ****P<0.0001

**Table S7 Relationship between LDHA expression and clinicopathological features of LSCC**

|  |  |  | **LDHA expression** | |  |
| --- | --- | --- | --- | --- | --- |
| **Parameter** | **Group** | **Total** | **Low** | **High** | **P value** |
| Age | <60 | 33 | 18 | 15 | 0.8352 |
|  | ≥60 | 27 | 14 | 13 |  |
| Gender | Male | 43 | 22 | 21 | 0.5920 |
|  | Female | 17 | 10 | 7 |  |
| T classification | T1/T2 | 33 | 20 | 13 | 0.2119 |
|  | T3/T4 | 27 | 12 | 15 |  |
| Lymph nodes | Positive | 24 | 6 | 18 | 0.0003  （***） |
|  | Negative | 36 | 26 | 10 |  |
| Clinical stage | Ⅰ,Ⅱ | 26 | 18 | 8 | 0.0309（*） |
|  | Ⅲ,Ⅳ | 34 | 14 | 20 |  |

*P < 0.05, **P < 0.01, ***P < 0.001, ****P<0.0001

**Table S8 Relationship between the level of lactate and clinicopathological features of LSCC**

|  |  |  | **The level of lactate** | |  |
| --- | --- | --- | --- | --- | --- |
| **Parameter** | **Group** | **Total** | **Low** | **High** | **P value** |
| Age | <60 | 33 | 15 | 18 | 0.4363 |
|  | ≥60 | 27 | 15 | 12 |  |
| Gender | Male | 43 | 23 | 20 | 0.3901 |
|  | Female | 17 | 7 | 10 |  |
| T classification | T1/T2 | 33 | 20 | 13 | 0.0693 |
|  | T3/T4 | 27 | 10 | 17 |  |
| Lymph nodes | Positive | 24 | 7 | 17 | 0.0084  （**） |
|  | Negative | 36 | 23 | 13 |  |
| Clinical stage | Ⅰ,Ⅱ | 26 | 18 | 8 | 0.0092  （**） |
|  | Ⅲ,Ⅳ | 34 | 12 | 22 |  |

*P < 0.05, **P < 0.01, ***P < 0.001, ****P<0.0001

**Additional methods:**

**Method S1**

Immunohistochemistry (IHC)：

The paraffin specimens were incised by 4 mm thick in human tissues. The immunohistochemistry analysis was performed with anti-LDHA antigen (dilution 1:800; #3582/clone C4B5) as described previously. The assessment of the immunohistochemistry staining was accomplished by two experienced pathologists with unified criteria and a single-blind method. The staining intensity was evaluated on a 4-step scale (0, no staining; 1, weak intensity; 2, moderate intensity; 3, strongest intensity). The fraction of stained cells was scored according to the following criteria. Score 0 (5% positive cancer cells), score 1 (5–25% positive cancer cells), score 2 (25–50% positive cancer cells), score 3 (50–75% positive cancer cells), and score 4 (75% positive cancer cells). The product of staining intensity and extent gave an overall staining score: negative = score 0; 1+ = score 1-4; 2+= score 5-8; 3+ = score 9-12. All the dyeing evaluation was got by the unified criteria and single-blind method. A score of 4 was used to distinguish between low (≤4) and high (>4) levels of LDHA gene expression.

**Method S2**

Western blot analysis

Proteins in LSCC cell lines (TU212,) were harvested in RIPA buffer and analyzed by western blot, as was described previously. Protein concentrations were quantified by the BCA protein quantification kit (KTD3001, Abbkine, CA, USA). Equal amounts of proteins (40 μg) were separated by SDS-PAGE, transferred to polyvinylidene difluoride (PVDF) membrane, and incubated with the following antibodies: anti-LDHA (dilution 1:1000; #3582/clone C4B5), anti-phospho-LDHA (dilution 1:1000; #8176), anti-β actin (dilution 1:1000; ab8226; Abcam Biochemicals, UK). β-actin expression was used as the loading control.
